# Unraveling the mechanism of recognition of the 3’ splice site of the adenovirus major late promoter intron by the alternative splicing factor PUF60

**DOI:** 10.1371/journal.pone.0242725

**Published:** 2020-11-30

**Authors:** Hsin-hao T. Hsiao, Gregg V. Crichlow, James W. Murphy, Ewa J. Folta-Stogniew, Elias J. Lolis, Demetrios T. Braddock

**Affiliations:** 1 Department of Pathology, Yale University School of Medicine, New Haven, Connecticut, United States of America; 2 Department of Molecular Biophysics and Biochemistry, Yale University School of Medicine, New Haven, Connecticut, United States of America; 3 Department of Pharmacology, Yale University School of Medicine, New Haven, Connecticut, United States of America; 4 W.M. Keck Biotechnology Research Laboratory, Yale University School of Medicine, New Haven, Connecticut, United States of America; Government College University Faisalabad, PAKISTAN

## Abstract

Pre-mRNA splicing is critical for achieving required amounts of a transcript at a given time and for regulating production of encoded protein. A given pre-mRNA may be spliced in many ways, or not at all, giving rise to multiple gene products. Numerous splicing factors are recruited to pre-mRNA splice sites to ensure proper splicing. One such factor, the 60 kDa poly(U)-binding splicing factor (PUF60), is recruited to sites that are not always spliced, but rather function as alternative splice sites. In this study, we characterized the interaction of PUF60 with a splice site from the adenovirus major late promoter (the *AdML* 3' splice site, *AdML*3’). We found that the PUF60–*AdML*3’ dissociation constants are in the micromolar range, with the binding affinity predominantly provided by PUF60’s two central RNA recognition motifs (RRMs). A 1.95 Å crystal structure of the two PUF60 RRMs in complex with *AdML*3’ revealed a dimeric organization placing two stretches of nucleic acid tracts in opposing directionalities, which can cause looping of nucleic acid and explain how PUF60 affects pre-mRNA geometry to effect splicing. Solution characterization of this complex by light-scattering and UV/Vis spectroscopy suggested a potential 2:1 (PUF60_2_:*AdML*3’) stoichiometry, consistent with the crystal structure. This work defines the sequence specificity of the alternative splicing factor PUF60 at the pre-mRNA 3’ splice site. Our observations suggest that control of pre-mRNA directionality is important in the early stage of spliceosome assembly, and advance our understanding of the molecular mechanism by which alternative and constitutive splicing factors differentiate among 3’ splice sites.

## Introduction

Pre-mRNA splicing is a critical mechanism of post-transcriptional processing required for the maturation of mRNA [1]. Alternative splicing adds complexity to the expression of many eukaryotic genomes by including or skipping various exons/introns [2–4]. Defects of pre-mRNA splicing have gained increasing notice due to its clinical implications in both diagnostics and therapeutics [5]. Examples include the pursuit of modifying the splicing of apolipoprotein B (APOB) pre-mRNA to lower circulating cholesterol levels and of survival of motor neuron 2 (SMN2) pre-mRNA for treatment of spinal muscular atrophy [6].

In eukaryotes, constitutive splicing is a dynamic multi-step process of spliceosome assembly involving small nuclear ribonucleoprotein particles (snRNPs), their auxiliary splicing factors, and many other RNA-binding proteins (RNABPs). The early-stage recognition of the 3’ splice site (ss) region of the pre-mRNA sequences is considered critical for exon/intron definitions and therefore the final products of splicing. The spliceosome assembly process begins with the U1 snRNP binding to the 5’ ss, and splicing factor 1 (SF1) recognizing the branch point sequence (BPS) near the 3’ ss to form the E’ complex. The E’ complex is then converted into the E complex by recruiting the heterodimeric U2 snRNP auxiliary factor (U2AF), with its small subunit U2AF35 recognizing the 3’ terminal AG and the large subunit U2AF65 recognizing the poly-pyrimidine tract (PPT) in between the BPS and 3’ terminal AG. Continuing spliceosome assembly then transitions through the A, B, and C complexes to complete the catalysis [1,4].

U2AF65 is a major component facilitating the recognition of the 3’ ss region and the bridging of multiple splicing factors. Recombinant U2AF65 alone is sufficient to reconstitute the splicing activity in cell extracts depleted of U2AF [7]. U2AF65 contains an N-terminal Arg-Ser repeat-containing (RS) domain, two central canonical RNA recognition motifs (RRMs) and a C-terminal degenerate RRM, now categorized as the U2AF homology motif (UHM). U2AF65 uses its two central RRMs to recognize the PPT and its C-terminal UHM to associate with SF1 [8]. The RS domain is associated with BPS, and part of the linker between the RS domain and the first RRM provides the interaction interface for association with U2AF35 [9]. The binding to the 3’ terminal AG by U2AF35 can help stabilize U2AF65 on weak PPTs [10]. U2AF65 interacts cooperatively with SF1 to contribute to the initial recognition of the BPS/PPT region [8]. Biochemical and structural analyses also suggest that U2AF65 structures the pre-mRNA to prime for subsequent spliceosome assembly and juxtapose reactive functional groups on the pre-mRNA [9,11].

**Fig 1:**
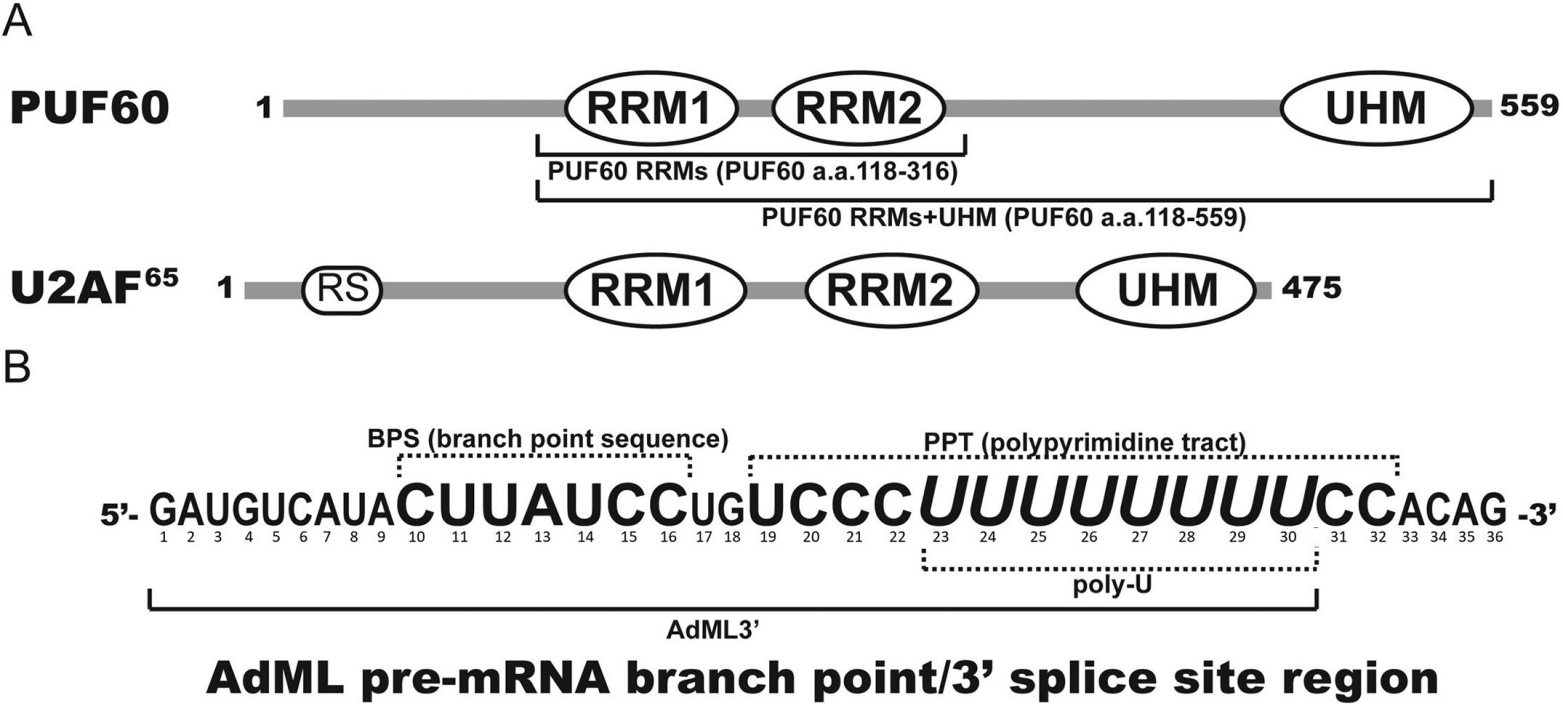
Domain structures of PUF60 and the *AdML* pre-mRNA 3’ intron sequence. (A) PUF60 is a 559-amino acid protein, consisting of two RNA-recognition motifs (RRMs) and one U2AF homology motif (UHM). PUF60 resembles U2AF65 in the two RRMs and one UHM, but lacks the N-terminal Arg-Ser repeat-containing (RS) domain that U2AF65 possesses to associate with the branch point sequence (BPS). (B) The sequence of the 3’-adenovirus major late promoter (*AdML3*’) pre-mRNA 3’ splice site (ss) region used in these studies [10,22]. *AdML3*’ contains the whole BPS, and the poly-U tract, which represents the majority of the polypyrimidine tract (PPT). The 3’ terminal AG splice site is positioned on the far right.

Homologous to both U2AF65 and the yeast Mud2p, the Poly(U)-binding splicing factor 60 kDa (PUF60) shares domain features with U2AF65 by also containing two central RRMs and a C-terminal UHM [12]. The most noticeable differences of domain structure between PUF60 and U2AF65 include the lack of the RS domain in PUF60 and a highly extended linker connecting the second RRM and UHM in PUF60 (Fig 1).

PUF60 can associate with either itself or U2AF65 [13,14]. PUF60 may function on some substrates to a degree in the absence of U2AF *in vitro*, but the presence of both proteins enhances splicing efficiency [14]. PUF60 and U2AF65 appear to co-facilitate the association of U2 snRNP with pre-mRNA [12]. It was proposed that U2AF65 and PUF60 may associate with the pre-mRNA in a sequential manner. U2AF65 may bind first to the pre-mRNA, and thereby recruit PUF60. The subsequent binding of PUF60 may weaken the association between U2AF65 and the pre-mRNA [14].

Although both U2AF65 and PUF60 are general splicing factors, the PUF60/U2AF65 ratio in cells influences alternative splicing patterns [14], suggesting PUF60 is implicated in alternative splicing. PUF60 appears to promote splicing at a weak 3’ splice site [12,14]. PUF60 is associated with the alternative splicing of the amyloid precursor protein (APP) minigene [13]. Depletion of PUF60 from HeLa cells favors brain-specific splicing for APP and bridging integrator 1 (BIN1). Mutation of the *Drosophila* ortholog of PUF60, Half pint (Hfp), alters alternative splicing patterns during development [15]. Deletion of PUF60 results in Verheij syndrome [16–18], a developmental disorder characterized by cardiac defects, microcephaly, short stature, intellectual disability and developmental delay, and often coloboma [19–21]. Mutations in the *puf60* gene also have been found to cause this syndrome [17,21].

To understand the structural basis behind the commonality and difference between the functions of PUF60 and U2AF65 in constitutive and alternative splicing, the functional and structural features of the core domains of PUF60 –two central RRMs and one C-terminal UHM—are delineated. Herein is reported a 1.95 Å crystal structure of the two RRMs of PUF60 (PUF60 RRMs) in complex with a sequence from the adenovirus major late promoter (*AdML*) pre-mRNA 3’ splice site region (*AdML*3’). The structure reveals a homo-dimeric organization, where each participating PUF60 monomer captures a nucleic acid tract, and the PUF60 dimer places two nucleic acid tracts in antiparallel directionalities. A solution study verifies that the nucleic acid binding affinity predominantly comes from its two central RRMs, with dissociation constants in the micromolar-range. Solution characterization of the complex by light-scattering and UV/Vis spectroscopy confirms the 3’ intron sequence-induced dimerization of PUF60 mediated by its two central RRMs. This binding behavior of PUF60 to the *AdML* 3’ intron sequence suggests an alternative strategy adopted by PUF60 to direct the conformation of the 3’ intron where the directionalities of the pre-mRNA would be similarly reversed as by U2AF65 [9,11], but with a unique loop generated. Similarly, as for U2AF65, a cooperative interaction also exits between PUF60 and SF1, in which interaction with PUF60 allocates its C-terminal UHM to associate with SF1. The more extended linker between the second RRM and the UHM of PUF60 compared to U2AF65 is likely conferring flexibility for UHM to locate SF1 on the pre-mRNA loop generated by PUF60 binding. Our observations suggest the importance of the modulation of the pre-mRNA directionality in the early stage of spliceosome assembly. The alternative “looping” strategy used by PUF60 may generate distinct local structures in the pre-mRNA 3’ss region, which may have implications in the transition of functional complexes during spliceosome assembly and/or intron/exon definitions.

## Materials and methods

### Cloning, protein expression, purification, and complex formation

Nucleotides encoding human Splicing Factor 1 (SF1) (UniProtKB/Swiss-Prot code Q15637) amino acids 1–260, were cloned into the pET100 protein expression vector with the Champion^™^ pET Directional TOPO^®^ Expression System (Invitrogen). Cloning artifacts introduced one glycine at the N-terminus of the protein following cleavage of a histidine tag with TEV protease. To ease protein handling, cysteine 171 was replaced by alanine (Cys171Ala) by QuickChange^®^ Site-Directed Mutagenesis Kit (Stratagene, La Jolla, CA).

Nucleotides encoding human PUF60 amino acids 118–316 (here referred to as PUF60 RRMs), were cloned into the pET15b protein expression vector with an R to G mutation at amino acid 106. After cleavage of a histidine tag with TEV protease, cloning artifacts introduced 16 amino acids (GHMASMTGGQQMGRGS) at the N-terminus of the protein, of which most are disordered and not visible in the final electron density. Nucleotides encoding human PUF60 (UniProtKB/Swiss-Prot code: Q9UHX1) amino acids 118–559 (here referred as PUF60 RRMs+UHM), were cloned into the pET100 protein expression vector. Cloning artifacts introduced 4 amino acids (GRGS) at the N-terminus of the protein following cleavage of a histidine tag with TEV protease. To ease protein handling, cysteine residues in both PUF60 sequences were replaced by serine (Cys129) or alanine (Cys255, Cys487) depending on their predicted location in the protein structure (alanine if buried, serine if exposed). All proteins were expressed in *Escherichia coli*, strain BL21 (DE3), using standard methods.

We used an oligonucleotide sequence (here referred to as *AdML*3’: 5’-GAUGUCAUACUUAUCCUGUCCCUUUUUUUU-3’, 30-mer) that corresponds to the 3’ intron sequence of the *AdML* splicing substrate [10,22], and encompasses the branchpoint sequence (5’-CUUAUCC-3’) and the full U-tract of the polypyrimidine tract (5’-UCCCUUUUUUUU -3’) (Fig 1). Synthetic *AdML*3’ RNA and the *AdML*3’ analogues (including (1) a DNA backbone that carries the *AdML*3’ AUCG base sequence, here referred to as dAdML3’; (2) 5’-fluorescein-labeled dAdML3’; (3) dAdML3’ brominated at carbon 8 of either G4 or G18) were purchased from The Midland Certified Reagent Company (Midland, TX).

### Size exclusion chromatography-coupled laser light scattering

The light scattering data were collected using a Superdex 200, 10/30, HR Size Exclusion Chromatography (SEC) column (GE Healthcare, Piscataway, NJ), connected to High Performance Liquid Chromatography System (HPLC), Agilent 1200, (Agilent Technologies, Wilmington, DE) equipped with an autosampler. The elution from SEC was monitored by a photodiode array (PDA) UV/VIS (UV) detector (Agilent Technologies, Wilmington, DE), differential refractometer (RI) (OPTI-Lab rEx Wyatt Corp., Santa Barbara, CA), static and dynamic, multiangle laser light scattering (LS) detector (HELEOS II with QELS capability, Wyatt Corp., Santa Barbara, CA). The SEC-UV/LS/RI system was equilibrated in 50mM Tris-HCl, 150mM NaCl, 20μM EDTA, pH 8.0, buffer at the flow rate of 1.0 ml/min. Two software packages were used for data collection and analysis: the Chemstation software (Agilent Technologies, Wilmington, DE) controlled the HPLC operation and data collection from the multi-wavelength UV/VIS detector, while the ASTRA software (Wyatt Corp., Santa Barbara, CA) collected data from the refractive index detector, the light scattering detectors, and recorded the UV trace at 280, 295 or 315 nm sent from the PDA detector. The weight average molecular weights, *Mw*, were determined across the entire elution profile in the intervals of 1 sec from static LS measurement using ASTRA software as previously described [23].

*Mw* for PUF60 RRMs+UHM:dAdML3’ complex was determined at a concentration range from 2 μM to 111 μM from 9 independent analyses of PUF60:dAdML3’ complexes, and from PUF60 RRMs+UHM alone from 7 μM to 115 μM from three analyses. The weight average molecular mass, *Mw*, for PUF60 RRMs:dAdML3’ complex was determined at concentration range from 4.6 μM to 169.3 μM from 10 independent analyses of PUF60:dAdML3’ complexes, and from PUF60 RRMs alone from 6 μM to 124 μM from four analyses. The samples were dilutions of PUF60:dAdML3’ complexes formed by mixing the protein and oligonucleotides at a 1:1.1 molar ratio in a buffer containing 50 mM Tris-HCl, 150 mM NaCl, 20 μM EDTA, pH 8.0.

### Fluorescence anisotropy analysis

Binding of PUF60 RRMs+UHM or RRMs to the RNA analogue was monitored by a change in the steady-state fluorescence anisotropy (r) of a 5’-fluorescein-labeled 30-mer sequence from the 3’ end of the *AdML* intron region (*5’-GAUGUCAUACUUAUCCUGUCCCUUUUUUUU-3’ on a DNA backbone, hereafter referred as *AdML3’). The concentration of *AdML3’ was kept constant at 10 nM, while the concentration of protein was varied from 0 to 200 μM. Samples were prepared in a buffer of 50mM Tris–HCl, 150mM NaCl, 20μM EDTA, pH 8.0, and equilibrated at room temperature for at least 30 min before measurements were taken on EnVision^™^ Multilabel Plate Reader—model 2101 (Perkin Elmer, Waltham, MA). Reactions were excited with a 480 nm polarized filter, and the emissions were read with two 535 nm polarized filters, one parallel to the polarization of the excitation filter, and the other perpendicular.

Equilibrium binding affinities were obtained by fitting the [PUF60]-dependent change of anisotropy (Δ*r*([PUF60])) to the following equation, which assumes that two PUF60 molecules bind sequentially to two independent sites on the *AdML3’:

$$r_{obs}-r_{o}=\Delta r(\left[PUF60\right])=\frac{\Delta r_{1}[PUF60]/K_{d1}+\Delta r_{2}{\left[PUF60\right]}^{2}/(K_{d1}K_{d2})}{1+[PUF60]/K_{d1}+{\left[PUF60\right]}^{2}/(K_{d1}K_{d2})}$$ (1)

where *r*_*0*_ is the anisotropy of free *AdML3’ nucleotides, Δ*r*_*1*_ is the difference between *r*_*0*_ and the anisotropy of one PUF60 molecule bound to *AdML3’, Δ*r*_*2*_ is the difference between *r*_*0*_ and the anisotropy of two PUF60 molecules bound to *AdML3’, *K*_*d1*_ is the equilibrium dissociation constant for the first PUF60 binding to *AdML3’, and *K*_*d2*_ is the equilibrium dissociation constant for the second PUF60 binding to *AdML3’.

### X-ray crystallography

#### Crystallization and data collection

Both PUF60 RRMs:dAdML3’ and PUF60 RRMs:brominated-dAdML3’ complexes were formed by mixing the protein and oligonucleotides at a 1:1.1 molar ratio in a buffer containing 50 mM Tris-HCl, 150 mM NaCl, 20 μM EDTA, pH 8.0. Crystals of the PUF60 RRMs:dAdML3’ complex grew from hanging drops at 20°C in at least 7 days after the protein solution (10 mg/ml PUF60 RRMs with nucleic acid, 50 mM Tris–HCl, pH 8.0, 150 mM NaCl, 20 μM EDTA) was mixed with an equal volume of the reservoir solution of 0.1 M Tris–HCl, 25% PEG 4000, 5–10 mM barium chloride dihydrate, pH 8.7. The same crystallization conditions were used for the complexes with the wild-type, and the two brominated dAdML3’ constructs (with either G4 or G18 brominated on carbon 8). Data using wild-type (unbrominated) dAdML3’ were collected at Advanced Photon Source Beam 24-ID-C. Data using the brominated constructs were collected at CHESS station A1. The crystals were flash frozen in liquid nitrogen without cryoprotection, mounted directly onto the beamline. Unbound PUF60 RRMs was crystallized using hanging drop vapor diffusion upon mixing 10 mg/ml protein with an equal volume of 0.1 M HEPES (pH 7.5), 1 M Li_2_SO_4_, 5% glycerol. X-ray data of the unbound protein were collected on a Rigaku rotating anode x-ray generator with an R-Axis IV detector. All data sets were processed using HKL2000 [24].

#### Solvent fraction calculation

The solvent content was calculated from the Mathews coefficient using the partial specific volume of the separate protein and RNA analogue constituents according to the method of Matthews as modified by Kantardjieff and Rupp [25,26].

#### Structure determination

Molecular replacement was performed with the program Phaser through the CCP4 [27,28] suite for the bound PUF60 RRM complexes and with Molrep [29] for the unbound structure. The molecular replacement was performed using protein coordinates of the published structure of the FIR:FUSE complex (PDB ID 2QFJ, or in the case of the unbound PUF60 RRMs, using an earlier, partially refined set of coordinates from the FIR:FUSE data). Nucleic acid coordinates were not included in any of the search models. The twinning fractions were found to be 0.49 (unbrominated), 0.46 (G4-brominated) and 0.47 (G18-brominated) using CNS [30,31]. The crystals were treated as perfect twins, and the data accordingly detwinned using CNS. Structure refinement was performed with REFMAC5 [32] and CNS. The native data set was refined to 1.95 Å using CNS. The data for the brominated complexes were similarly detwinned, and the structures refined, using CNS (S1 Table). Manual adjustments were performed using the programs O [33] and COOT [34]. Crystallographic software was licensed through the SBGrid [35].

### Isothermal titration calorimetry

The heat generated by addition of SF1 1–260 to PUF60-RRMsUHM or RRMs in a sample cell was measured at 30°C using a VPITC calorimeter (Microcal, Piscataway, NJ). Samples were extensively dialyzed into 50 mM NaCl, 25 mM Hepes, (pH 7.4) before experiments. The heat from the last 10 injections of each experiment were similar and the average of them was subtracted from the whole dataset for correction for enthalpy of dilution. The PUF60-RRMsUHM datasets were further corrected by the subtraction of the reference titration of SF1 enthalpy of dilution. Data were processed and thermodynamic parameters obtained using the least squares fitting routines available in the Origin v5.0 software (Microcal, Piscataway, NJ).

### Electrophoretic mobility shift assay

Proteins were mixed with RNA oligonucleotides (5μM final concentration in 20μl total reaction volume) in 50 mM Tris-HCl, 150 mM NaCl, 20 μM EDTA, pH 8.0. True RNA was used for this assay (*AdML*3’); a deoxyribose backbone was not employed. Ten microliters of 6X Phenol Blue dye was added to the reactions prior to sample loading. Electrophoresis was performed in 0.5X TBE 5.5% native polyacrylamide gels run at 300V for 120 minutes at 4°C. To denote the relative locations of band shifts, 3μl of 1kb+ DNA ladder was subjected to electrophoresis along with the samples. The gel was post-stained with SYBR Green II RNA gel stain (Invitrogen, Carlsbad, CA) in the cold room for 40 min before visualization under SYBR^®^ photographic filter.

## Results

### *AdML* 3’ splice site region sequence recognition by PUF60

To study the interaction between PUF60 and the *AdML* 3’ splice site region sequence, first the dissociation constants between a 30-mer analogue of *AdML*3’ (Fig 1) and two PUF60 constructs were determined by fluorescence anisotropy.

The *AdML* 3’ splice site region sequence is based on a biologically-relevant splicing substrate previously used [10,22]. The 30-mer fragment selected for this binding study contains the BPS, with flanking sequences on both ends. On the 3’ end to BPS, all the uridines of the PPT were included because uridine nucleotides were previously identified to be the key determinant for PUF60 recognition [12]. The oligonucleotide analogue used for the binding study was 5’ fluorescein-labeled, and synthesized with adenosine, uridine, cytosine, and guanidine-based deoxyribonucleotides (*AdML3’) to mimic the pre-mRNA, yet maintain stability. The PUF60 constructs used contain either all its RRMs and UHM (PUF 60 RRMs+UHM), or the RRMs only (PUF60 RRMs).

**Fig 2:**
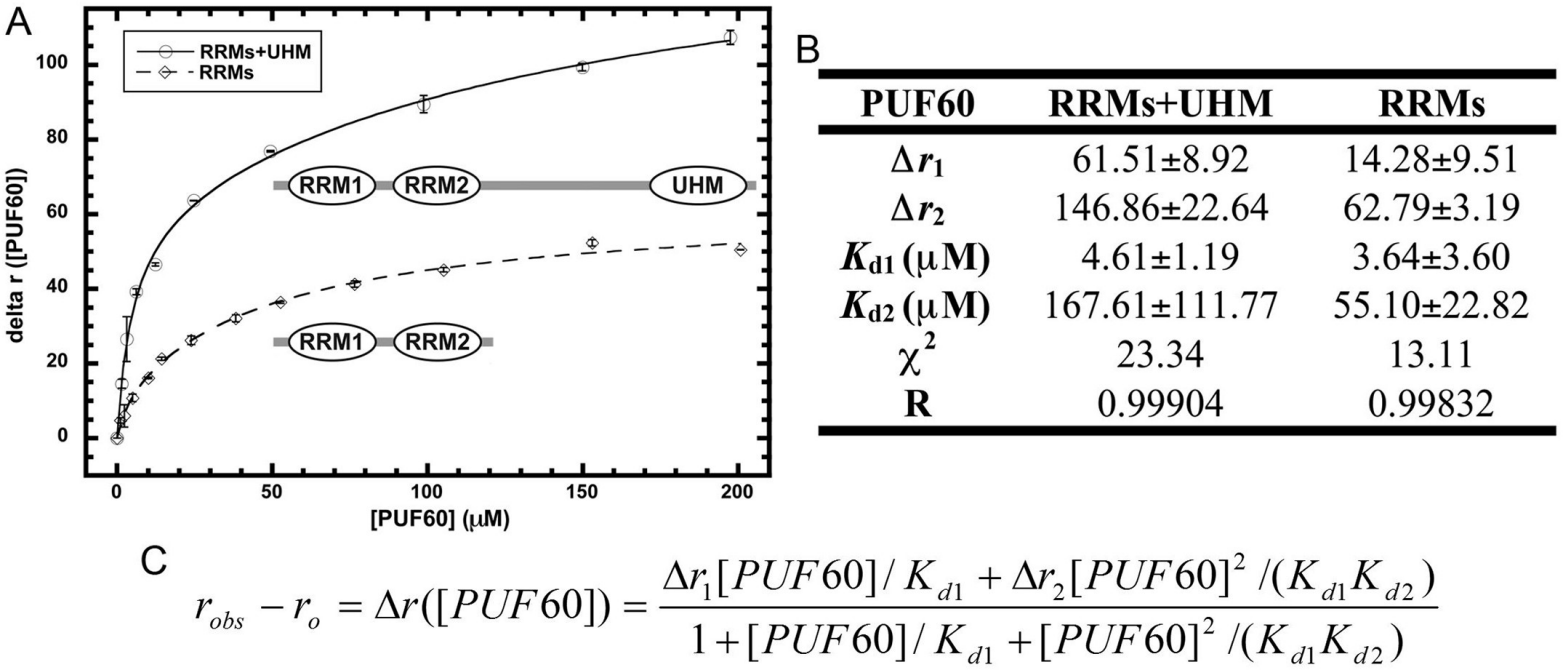
Binding of PUF60 domains to *AdML* pre-mRNA 3’ splice site region sequence. (A) Binding curves of PUF60 domains, RRMs+UHM or RRMs, to *AdML3’ as determined by fluorescence anisotropy. (B) Reported parameters of RRMs+UHM and RRMs binding to *AdML3’ by curve fitting with a 2:1 stoichiometric model. (C) Fitting equation assuming two-site sequential binding of two PUF60 molecules to one *AdML3’.

The binding curve (Fig 2(A)) for PUF60 constructs and *AdML3’ was fit to a two-site sequential binding model (Fig 2(C)) in accordance to a dimerizing behavior previously observed [36]. The dissociation constants for *AdML3’ are determined for PUF60 RRMs+UHM (K_d_1 = 4.61±1.19 μM; K_d_2 = 167.61±111.77 μM) and RRMs (K_d_1 = 3.64±3.60 μM; K_d_2 = 55.10±22.82 μM) (Fig 2(B)). RRMs+UHM and RRMs have comparable affinities toward *AdML3’. This observation suggests that the two central RRMs are the major domains responsible for *AdML3’ recognition.

### Structure of PUF60 RRMs in complex with *AdML* pre-mRNA 3’ splice site region sequence analogue

To understand the structural basis of the recognition between PUF60 and the pre-mRNA 3’ splice site region sequence, a crystal structure of the PUF60 RRMs in complex with the *AdML*3’ oligonucleotide analogue (with the deoxyribonucleotide backbone, but without the fluorescent label—dAdML3’) was determined at 1.95 Å (PDB ID: 5KVY). Use of the deoxyribose backbone was useful for enhancing stability of the nucleic acid, as protein crystallization can take many days to weeks, and it was important for the nucleic acid to remain stable for such a long period. RNA often is degraded by contaminating ribonucleases during such a long time frame. Structure determination was facilitated by molecular replacement based on our previously published coordinates of the same protein construct in complex with the FUSE DNA at 2.1 Å resolution (PDB ID: 2QFJ). Only protein coordinates were used in the molecular replacement. FUSE DNA binds to an alternatively spliced version of PUF60, known as FIR, which is identical in its RRMs. Statistics of crystallographic data and refinement are listed in S1 Table.

**Fig 3:**
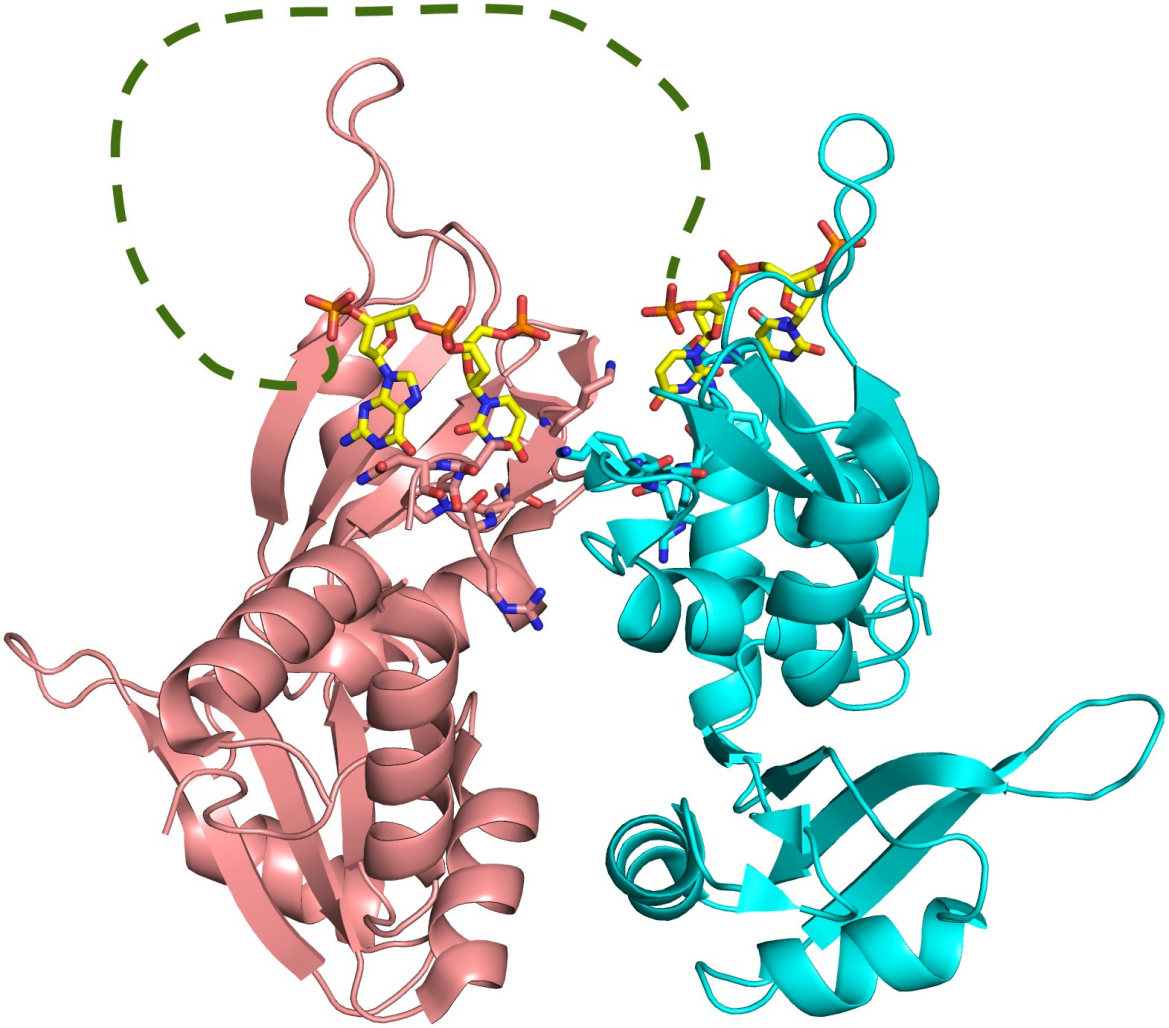
Overview of PUF60:dAdML3’ complex structure and its dimeric interface. The overall structure of PUF60 RRMs:dAdML3’ complex is analogous to that of the FIR:FUSE complex (PDB ID: 2QFJ) in many structural features including the dimeric conformation of RRMs. Subunit A of PUF60 RRMs is shown in pink, and subunit B in cyan. The bound dAdML3’ is shown with carbon atoms in yellow. The opposing 5’– 3’ directionalities of the observed nucleotides indicate that the backbone loops out as it extends from one side of the dimer to the other. A possible path of the nucleotide backbone is shown in green.

The overall structure of PUF60 RRMs:dAdML3’ complex is analogous to that of the FIR:FUSE complex (PDB ID: 2QFJ) in many structural features. First, there is a dimeric arrangement of RRMs. Second, each monomer only binds to a short tract of nucleic acid using only the RRM1 but not the RRM2. Third, the conventional residues used for RNA binding in RRM2 are buried in the interface between RRM 1 and RRM2. Fourth, the two helices in the linker between RRM1 and RRM2 form a cross-like structure, stabilizing the relative orientation of RRM1 and RRM2 (Fig 3).

Similar to what was observed in the FIR:FUSE DNA structure [36], there are two nucleotide tracts captured by the two RRM1 domains of the two protein chains in the dimeric structure. From the electron density, the directionalities of the two tracts can be confidently assigned, which are anti-parallel relative to each other. Each oligonucleotide tract extends its 3’ terminus toward the linker between RRM1 and RRM2 (Fig 3).

**Fig 4:**
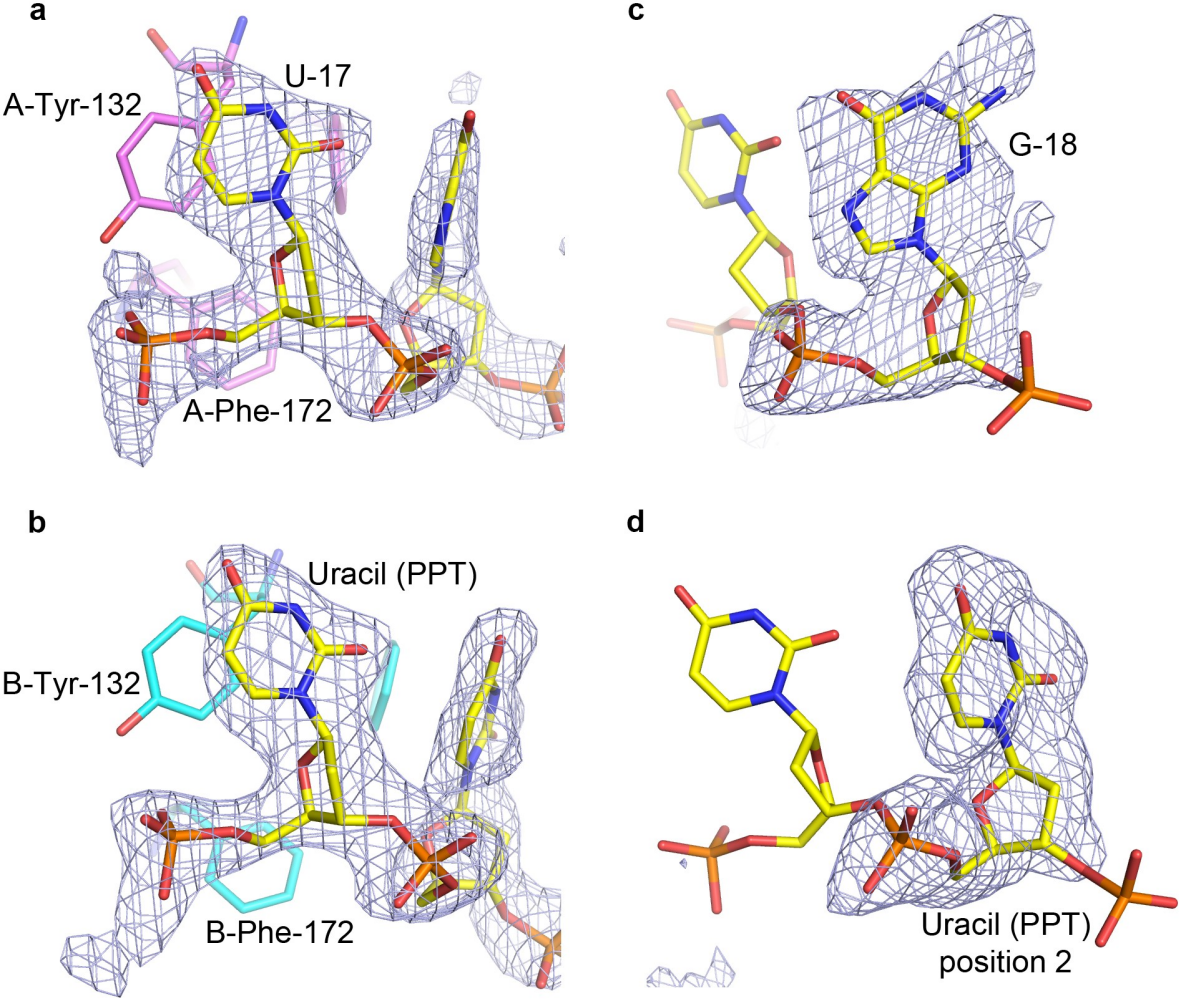
Identification of bound nucleotides. Simulated-annealing omit maps are displayed, omitting all nucleotides (A,B) or only the nucleotides in the second binding site on each subunit (C,D). The identification of the first nucleotide on each site allowed for that to be included in map calculations, and the second base became apparent in the electron density maps. (A) The uracil in the first binding site on subunit A (U-17). (B) The uracil (from the poly-U tract) in the first binding site on subunit B. (C) A guanine (G-18) is bound in the second binding site on subunit A. (D) However, a uracil is found in the second site on subunit B. The color-coding of chains in all panels is the same as in Fig 3.

**Fig 5:**
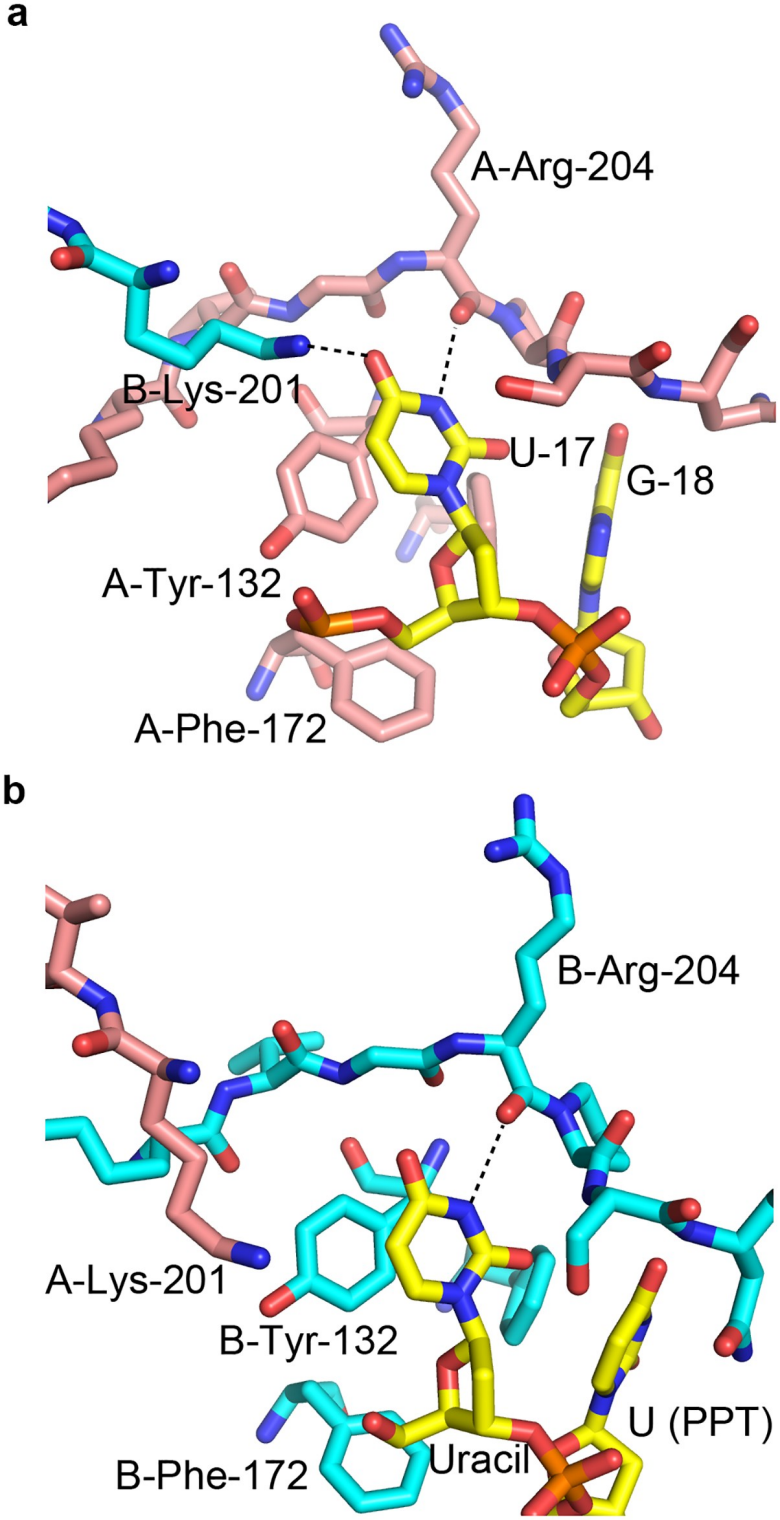
Recognition of bases bound in the first position of the nucleic acid binding site on the PUF60 RRM domains. (A) U-17 (immediately after the BPS) bound to subunit A. Lys-201 from subunit B donates a hydrogen bond to the nucleotide. (B) Poly-U tract uracil bound to subunit B.

Two bases can be seen in the electron density bound to each subunit. The first base on each side is clearly observed as uracil (Fig 4). Although uracil and cytosine are isosteric and would look identical in the electron density, the hydrogen bonding distinguishes uracil from cytosine. The base in the first position on each side donates a hydrogen bond from the nitrogen at position 3 to the main chain carbonyl of Arg-204 of the proximal PUF60 subunit (Fig 5). If the base were a cytosine, the atom at position 3 would be a deprotonated nitrogen, and would not be able to donate a hydrogen bond. Uracil, however, has a protonated nitrogen at this position, which is able to donate the hydrogen bond. Therefore, the first base on each subunit must be uracil. Furthermore, the first position uracil bound to subunit A accepts a hydrogen bond from Lys-201 of subunit B (Fig 5), as discussed in the next section. RRM1 residues surrounding the uridine are shown in Fig 5, where Tyr-132 is the most outstanding feature that provides stacking for the uracil base. Phe-172 and Phe-174 may participate to define the shape of the binding pocket. Phe-174 also provides stacking for the base in the second position.

**Fig 6:**
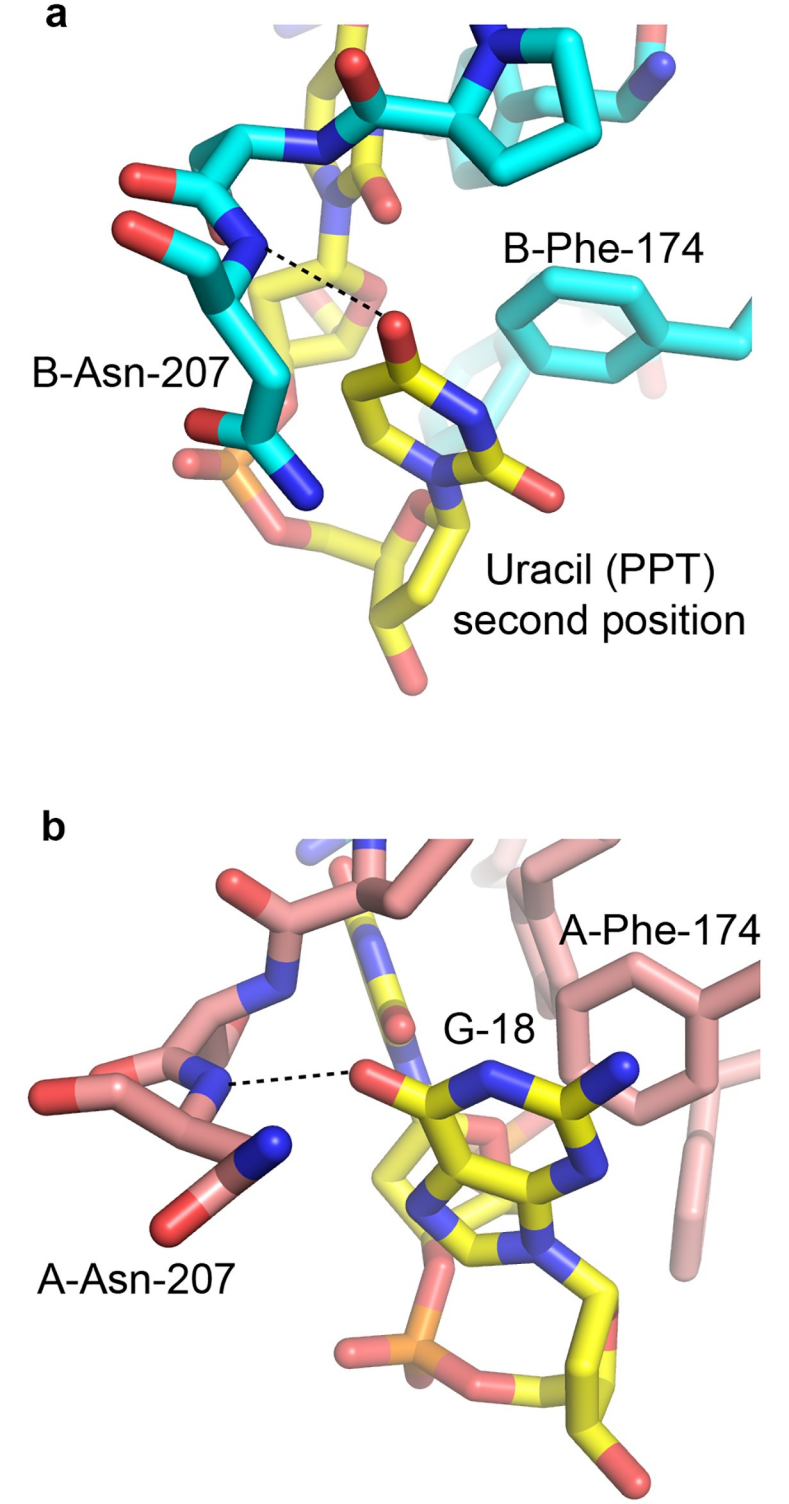
Recognition of second position bases by PUF60 RRMs. Hydrogen-bonding helps identify the identity of bases bound to the second nucleotide binding site on each PUF60 RRM subunit. (A) A uracil in the poly-U region accepts a hydrogen bond from Asn-207 of subunit B in the second nucleotide binding site. (B) The equivalent Asn-207 of subunit A donates a hydrogen bond to G-18 in the second nucleotide position on that particular subunit.

The density for the next base is different on each subunit. For the second nucleotide base bound to subunit B, although the density is fairly weak, it is sufficient enough to show that it is a pyrimidine (Fig 4). The hydrogen bonding indicates that it is uracil based on the fact that the 4-carbonyl group is in hydrogen-bonding distance to the main chain amide of Asn-207 of subunit B (Fig 6). Asn-207 is in a region of high mobility or disorder, and therefore can likely adapt to accommodate either a purine or a pyrimidine. However, this would not favor a cytosine, which has an amino group at position 4 that cannot accept a hydrogen bond. A table of hydrogen bond distances are presented in S2 Table. All of the protein-nucleic acid hydrogen bonds involve the bases of dAdML3’. The nucleic acid backbone is not involved in any hydrogen bond interactions. This indicates that the use of a deoxyribose backbone in the construct is sufficient to provide information about the interaction between PUF60 and the RNA bases of the target sequence. The major nucleic acid-protein interactions are the aromatic interactions with Tyr-132, Phe-172 and Phe-174.

The electron density of the second base bound to subunit A is ambiguous; however, it is extensive enough to indicate that it is a purine (Fig 4). Again, the hydrogen bonding pattern was used to determine whether this is an adenine or a guanine. Asn-207 of subunit A donates a hydrogen bond to the substituent at position 6 of the purine (Fig 6). This shows that the base must be guanine, which has a carbonyl at position 6 that can accept a hydrogen bond, as opposed to adenine, which has an amino group at this position that would rather donate a hydrogen bond. Therefore, UG is found bound to one subunit and UU bound to the other.

There are two UG pairs in the oligonucleotide used in the co-crystallization—bases 3–4 and bases 17–18. In order to determine which UG is bound to the protein, two other oligonucleotides were used having sequences identical to dAdML3', but with either G4 or G18 brominated on carbon 8. Each of these was co-crystallized with PUF60 RRMs, and the structure determined at 2.10 Å (dAdML3'-BrG4, PDB ID: 5KW1) and at 1.90 Å (dAdML3'-BrG18, PDB ID: 5KW6). The structure of dAdML3'-BrG4 bound to PUF60 RRMs was essentially identical to that of wt dAdML3' bound to PUF60 RRMs (S1 Fig). No bromine atom was visible in the electron density, although the guanine was visible in the second position bound to subunit A. In contrast, the structure of the dAdML3'-BrG18 oligonucleotide bound to PUF60 RRMs revealed UU bound to both subunits of the dimer (S2 and S3 Figs), suggesting that the guanine that is bound in the wild-type sequence is G-18, but this cannot bind when it is brominated, presumably due to perturbation of the phosphodiester backbone—needed to avoid steric clash of the bromine atom with the backbone—which would not favor binding. Therefore, the bases of the wild-type sequence bound to the protein were assigned as U17-G18 on one side of the dimer, and UU from the polypyrimidine tract (PPT) on the other. The only other UU in the sequence consists of bases 11–12. However, there are not enough nucleotides between U-12 and U-17 to loop around from one side of the dimer to the other. Therefore, the looping in the wt dAdML3’ is deduced to occur between U17-G18 and the PPT. It is interesting to note that U17-G18 is found immediately after the BPS, and just before the PPT.

It is quite possible that the specific nucleotides bound in the poly-U sequence vary, and that the oligonucleotide can slide from one portion of the poly-U region to another. There is residual density observed after the nucleotide in the second position of the UU bound to subunit B, but the density is too poor to model the nucleotide. Therefore, in the deposited coordinates, the UU was labeled as U28-U29, although it may be a variety of consecutive UU bases in the poly-U tract.

**Fig 7:**
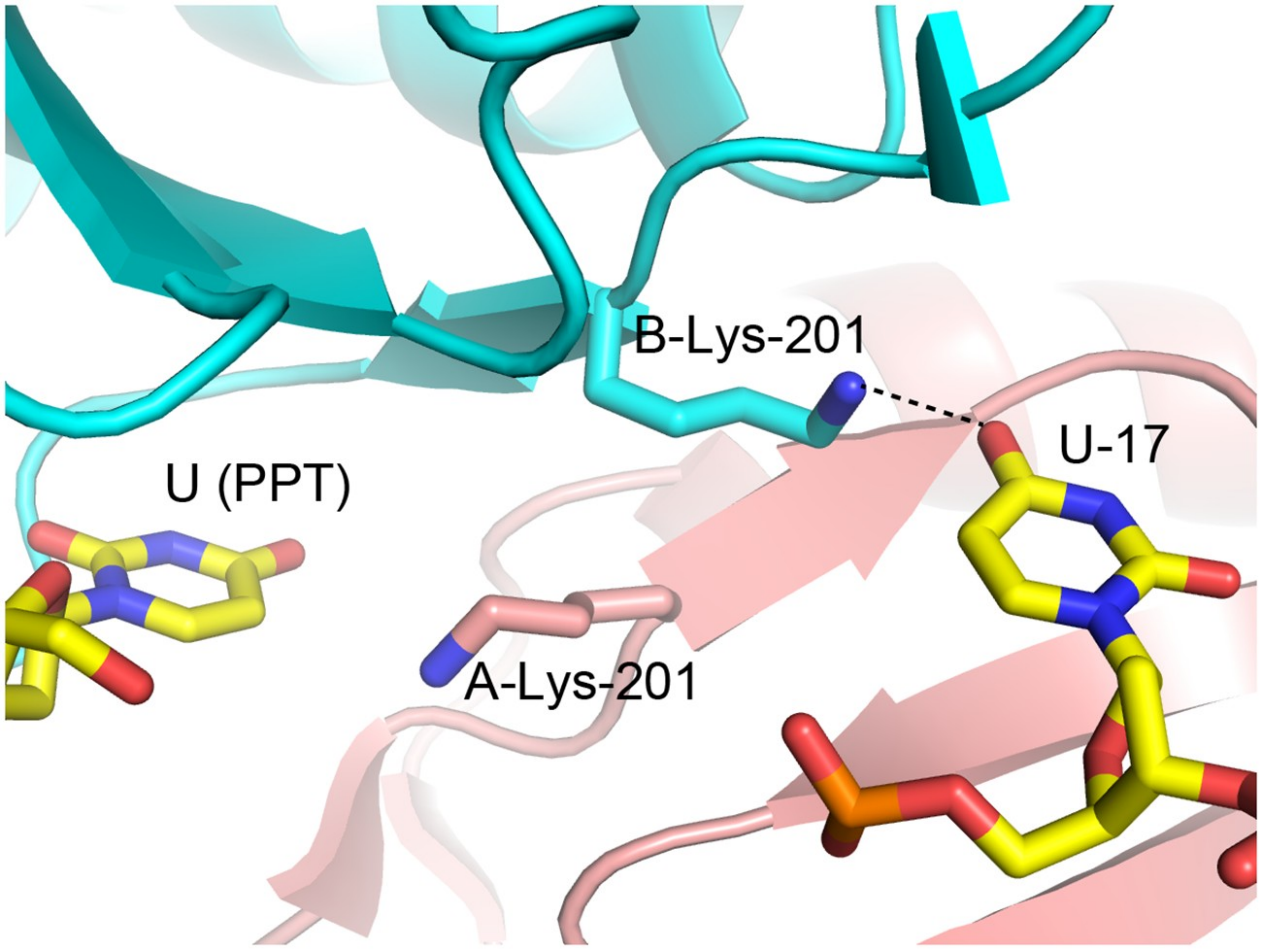
K201 residues are asymmetrically positioned, reaching across the dimerization interface to associate with bound nucleotides. Nucleotides and the K201 residues from both the A and B-chain monomers of PUF60 are shown as sticks. The rest part of the PUF60 structure is shown in ribbon-style. One of the Lys-201 residues donates a hydrogen bond to U-17 of dAdML3’ on the other side of the dimeric interface.

Lys-201 is another PUF60 residue which does not only associate with the uridine bound, but also crosses the dimerization interface and reveals asymmetrical positioning (Fig 7). The Lys-201 side-chain from subunit B contacts U17 of dAdML3’, which is bound on the other side of the dimeric interface. This lysine donates a hydrogen bond to the 4-carbonyl oxygen of U17. The side chain of Lys-201 from chain A approaches the 4-carbonyl oxygen of the PPT uracil bound in the first binding site near chain B, but does not approach closely enough to be considered a hydrogen bond donor. The oppositely facing orientations of the two Lys-201 residues could be due to their mutual repulsion from each other, and the attraction provided by the negative charge on the oligonucleotide backbone. Possibly, this asymmetry can form in either direction in solution. The interactions between the Lys-201 residues and the uridines across the PUF60 dimerization interface contributes to the dimerization upon recognition of the pre-mRNA sequence by PUF60.

The dimerization interface is similar to two hands coming together face-to-face, with the palms curving away from each other (Fig 3). The volume between these ‘palms’ are filled with water, and therefore this portion of the molecule does not contribute to the dimerization interface. All of the residues involved in the subunit-subunit interaction are polar, with hydrogen bonds stabilizing the interaction (S4 Fig).

### *AdML*3’ induces the dimerization of PUF60 RRMs

**Fig 8:**
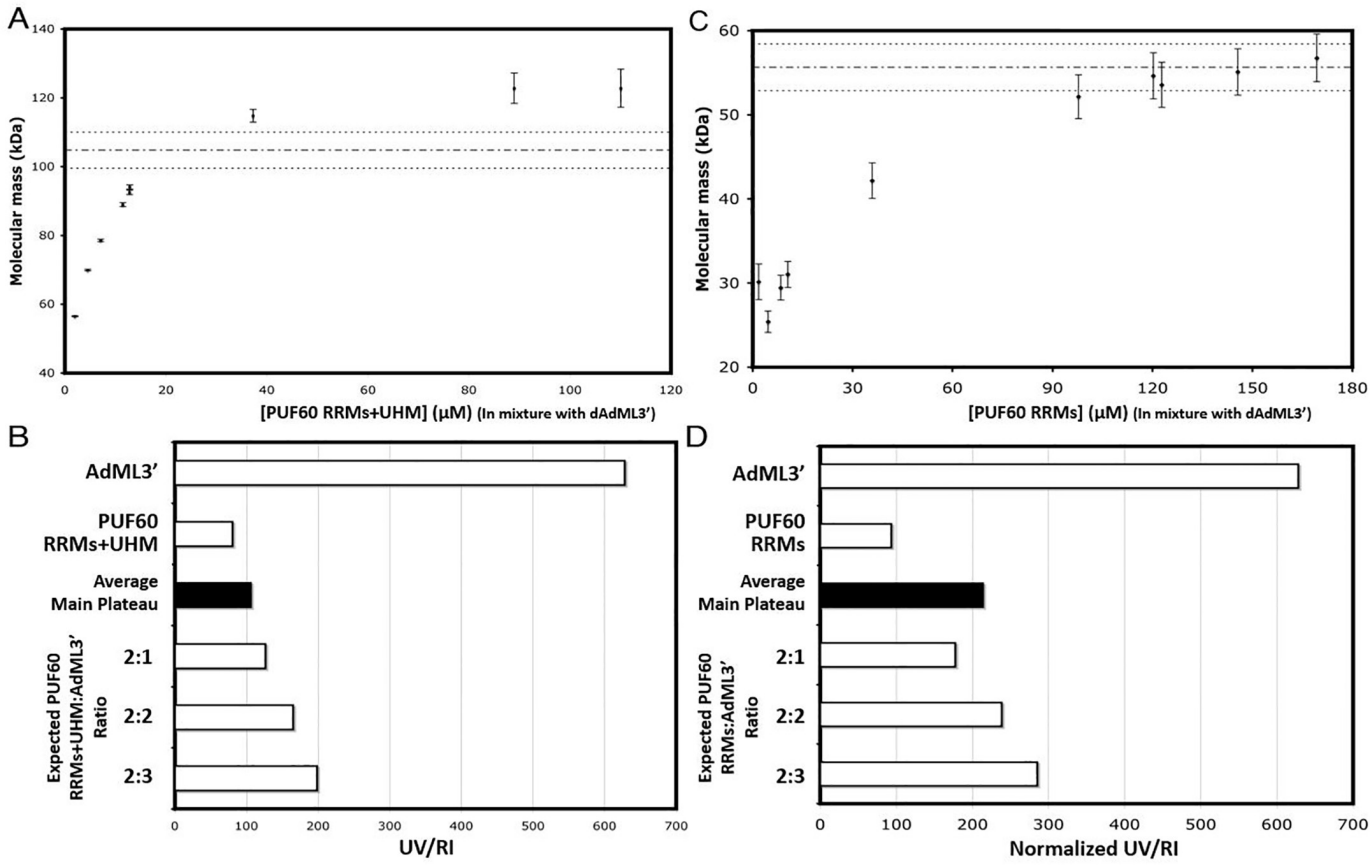
PUF60 dimerizes upon dAdML3’ binding. (A) SEC-LS analysis of the dAdML3’:PUF60 RRMs+UHM complex. Each data point represents an average molecular weight measured at the apex of an eluent peak of the complex at a certain concentration. Expected MW (104.8 kDa) for a 2:1 (protein: Nucleic acid) complex is indicated by the dashed line, with its expected 5% deviation shown in dotted lines; (B) UV/RI analysis of the dAdML3’:PUF60 RRMs+UHM complex. The UV/RI ratios from the three data points at the highest concentrations from (A) are averaged, and shown in the filled bar to be compared to other standard and expected values as described; (C) SEC-LS analysis of the dAdML3’:PUF60 RRMs complex. Each data point represents an average molecular weight measured at the apex of an eluent peak of the complex at a certain concentration. Expected MW (55.6 kDa) for a 2:1 (protein: Nucleic acid) is indicated by the dashed line, with its expected 5% deviation shown in dotted lines; (D) UV/RI analysis of the dAdML3’:PUF60 RRMs complex. The UV/RI ratios from the three data points at the highest concentrations from (C) are averaged, and shown in the filled bar to be compared to other standard and expected values as described.

In order to validate the stoichiometry of PUF60:dAdML3’ in solution, size-exclusion chromatography-coupled light scattering (SEC-LS) was used to analyze mixtures of PUF60 constructs with excess dAdML3’ (molar ratio of dAdML3’:PUF60≥1.1:1). Experiments were conducted under identical near-physiological conditions as previously used for the apo-protein [36]. Our current data shows that PUF60 RRMs+UHM gives rise to a plateau molecular weight (M.W.) above that of the dimer but less than that of trimer (Fig 8A). The UV/RI ratio of the PUF60 RRMs+UHM:dAdML3’ complex suggests the protein may aggregate onto dAdML3’ (Fig 8B). Analysis of the PUF60 RRMs:dAdML3’ complex reveals that the M.W. approaches the mass of a dimeric protein complexed to single-stranded dAdML3’ (Fig 8C). The UV/Vis ratio of the complex lies between the ones expected for 2:1 and 2:2 protein: nucleic acid stoichiometries (Fig 8D). However, in the absence of nucleotide, PUF60 RRMs does not dimerize [36], and neither does PUF60 RRMs+UHM (S5 Fig).

In summary, our data indicate that *AdML*3’ may induce the dimerization of PUF60 RRMs just as FUSE DNA induces the dimerization of FIR [36].

#### PUF60 and SF1 cooperative recognition of the 3’ splice site region of *AdML* pre-mRNA

U2AF65 utilizes its UHM to cooperatively interact with SF1 to facilitate BPS/PPT recognition [8]. In order to know whether a similar behavior also exits between PUF60 and SF1, the association between PUF60 and SF1 was studied by isothermal titration calorimetry (ITC), and also the ternary interactions between PUF60, SF1, and the *AdML* 3’ intron pre-mRNA sequence by electrophoretic mobility shift assay (EMSA).

**Fig 9:**
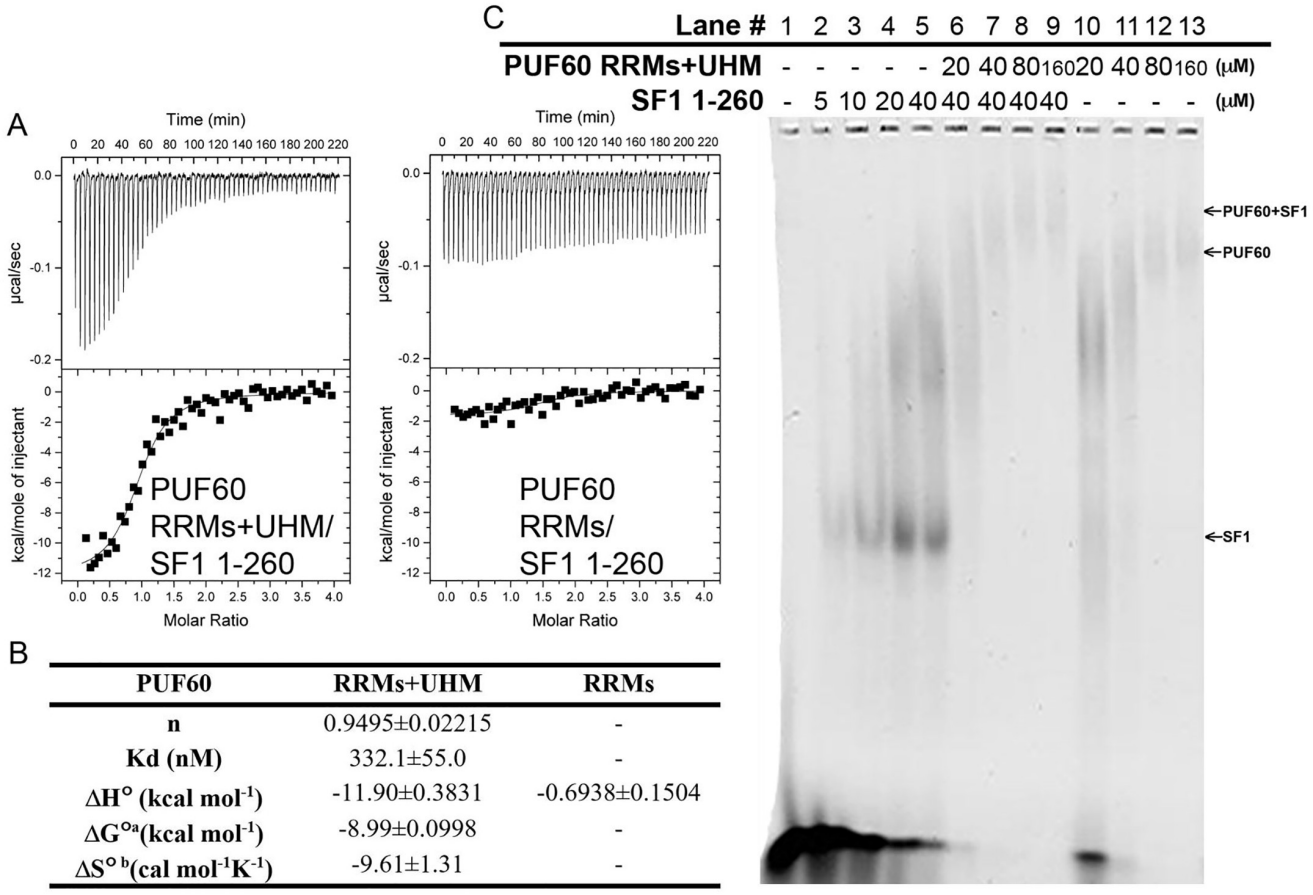
PUF60 uses UHM to associate with SF1 and cooperatively interact with *AdML*3’ splice site region pre-mRNA. (A) Representative isotherms from ITC experiments to detect the association between SF1 1–260 and either PUF60 RRMs+UHM or PUF60 RRMs. (Left panel) Titration of SF1 1–260 into PUF60 RRMs+UHM. The best fit of the binding stoichiometry (n) was one. All titrations were measured at 30°C. (Right panel) Titration of SF1 1–260 into PUF60 RRMs; (B) Reporting the thermal dynamic parameters measured in (A). The mean parameters and standard deviations of two experiments are reported. The standard state (°) for these experiments is defined as 1 M concentrations of each protein at 30°C. The dissociation constant (K_d_) and stoichiometry of binding (n) are derived from curve fitting based on datasets subtracted with the reference titration of SF1 heat of dilution. ^a^ Calculated using the equation ΔG° = –RT ln(K_d_^-1^). ^b^ Calculated using the equation ΔG° = ΔH°-TΔS°. (C) EMSA study of the interplay of SF1 1–260 and PUF60 RRMs+UHM with *AdML*3’ RNA. The bright-dark colors of the gel image are digitally reversed for clarity in presentation.

The protein-protein interaction between PUF60 RRMs+UHM and SF1 (residues 1–260) was characterized. ITC analysis (Fig 9A, left panel) reveals a dissociation constant of 332±55 nM (Fig 9B), while for PUF60 RRMs, there is no observed association with SF1 (Fig 9A, right panel).

EMSA was then used to study the interactions between the *AdML*3’ pre-mRNA and either SF1 or PUF60 RRMs+UHM individually and with both proteins. The far-left 5 lanes in Fig 9C, demonstrate that increasing SF1 1–260 concentrations up to 40 μM correlate with the increased amount of *AdML*3’ pre-mRNA bound. The far-right four lanes demonstrate that PUF60 RRMs+UHM also shifted the free RNA. Two conformations may exist for the PUF60 RRMs+UHM:*AdML*3’ pre-mRNA complex at 20 μM of PUF60 which then gradually converge into a larger complex as PUF60 concentration increases, consistent with the SEC-LS data. In the center four lanes of Fig 9C, the ternary interactions between the RNA and both SF1 and PUF60 were studied. When both proteins are present, all bound populations shift farther upward than when only one protein is present in the reaction, which suggests the presence of ternary complex of PUF60, SF1, and RNA, similar to what was observed for U2AF65, SF1, and RNA [8].

## Discussion

### Dimerization of PUF60 suggests an alternative strategy to structure the 3’ splice region of pre-mRNA

**Fig 10:**
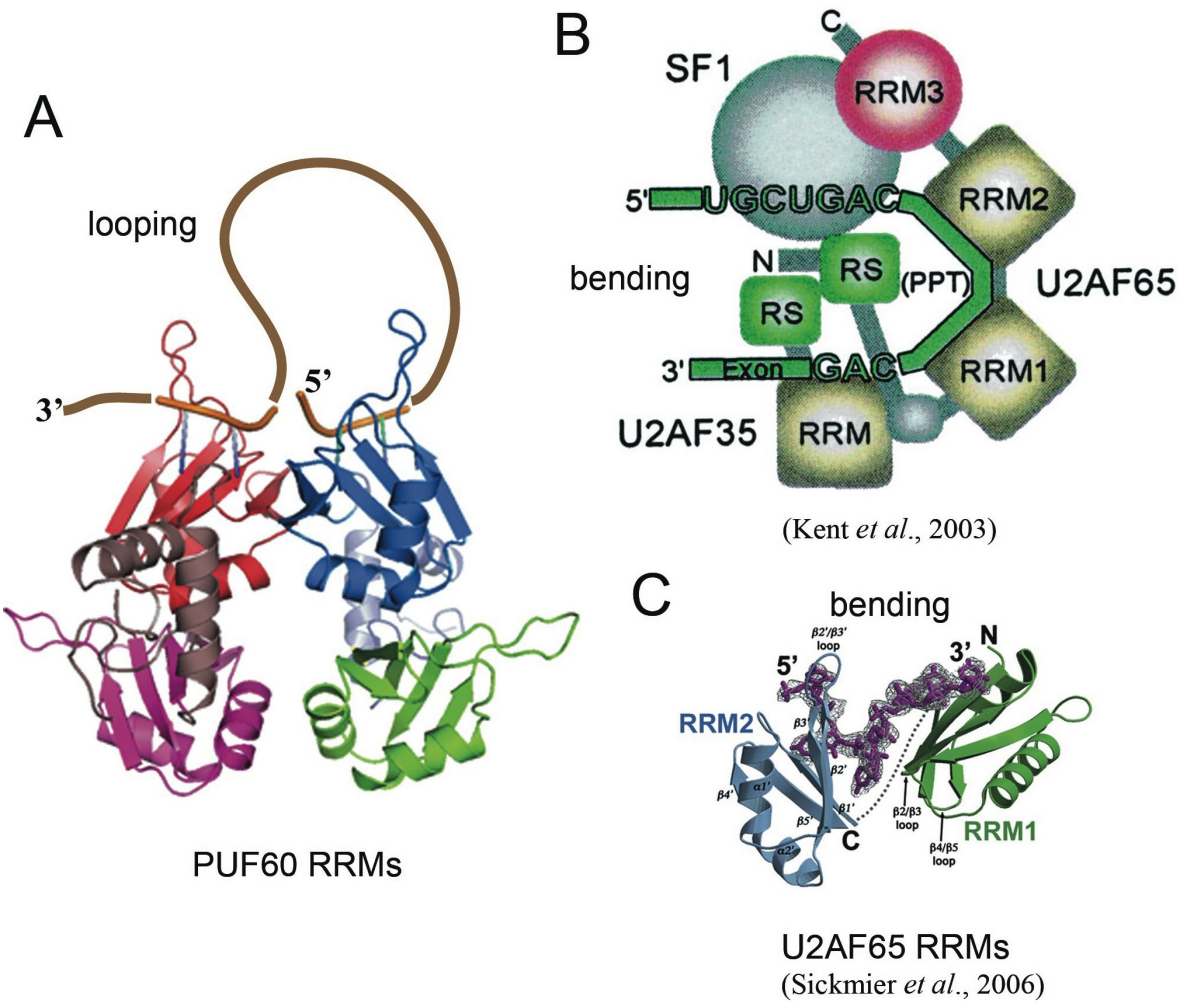
Alternative strategies to structure the 3’ spice site region of pre-mRNA: Looping by PUF60 vs. Bending by U2AF65. (A) Hypothetical drawing of the potential looping that can occur on pre-mRNA structure upon PUF60 binding; (B) Direct excerpt from the work of Kent *et al*. to show the current model for the bending of the pre-mRNA by U2AF65 and other factors [9]; (C) Direct excerpt from Sickmier *et al*. of the crystal structure of the U2AF65 RRMs in complex with a poly-U tract to demonstrate the bending mechanism supported by structural data [11]. Notice that, with either strategy, the directionality of the pre-mRNA will be modulated. The modulation of the directionality may bring distant components on the pre-mRNA into proximity.

The current model for U2AF65:pre-mRNA interaction suggests that U2AF65 may bend the 3’ ss region to prime the pre-mRNA for spliceosome assembly and juxtapose reactive functional groups (Fig 10(B)) [9,11]. The structure of dimeric PUF60 RRMs in complex with the *AdML* pre-mRNA 3’ ss region sequence suggests that PUF60 may also structure the pre-mRNA, albeit using a “looping” mechanism instead of “bending” as used by U2AF65 (Fig 10). Both strategies used by PUF60 and U2AF65 to structure the pre-mRNA would change the directionality of the pre-mRNA. This common outcome facilitated by either PUF60 or U2AF65 suggests that modulation of the directionality of the pre-mRNA is an important step in the early-stage 3’ ss recognition and spliceosome assembly.

The difference in the resulting pre-mRNA structure as manipulated by either PUF60 or U2AF65 is that a loop would be generated by PUF60 binding. Whether the loop structure has implications in the different functionality or the cooperative action between PUF60 and U2AF65 is a pending question. PUF60 can associate with U2AF65 [13,14], cooperating with it to associate U2snRNP with pre-mRNA [12]. It was proposed that PUF60 and U2AF65 may bind to pre-mRNA in a sequential manner, in which U2AF65 may bind first to the pre-mRNA, and recruit PUF60. Later, PUF60’s presence on the pre-mRNA may destabilize U2AF65 from the 3’ss region [14]. Does the bending by U2AF65 help shape the pre-mRNA for enhanced PUF60 binding, while the looping facilitated by the dimerization of PUF60 eventually weaken U2AF65 binding? The answer for this question may reveal an important mechanistic process allowing for transitioning through various stages of functional complexes in pre-mRNA splicing.

Solution studies with SEC-UV/LS/RI demonstrated that the presence of single stranded nucleic acid induced dimerization of PUF60 (both the RRMs+UHM and RRMs constructs), while the apo-PUF60 stayed monomeric even at high concentrations (S5 Fig) [36]. The PUF60 RRMs dimeric structure, although partially driven by the high concentration and/or lattice packing in the crystalline environment, possibly reveals a low-energy architectural state typically induced by PUF60-nucleic acid interactions at physiological concentrations. With no known literature of *in vivo* study on the conformation and stoichiometry of the functional PUF60 assemblies in cells, our crystal structure presents one of the best-supported models to explain how PUF60 might structurally impact the topology of substrate RNA to drive alternative splicing. The topology of the protein-RNA complex induced by protein dimerization would necessarily loop a single bound nucleic acid strand, and reverse pre-mRNA directionality at the 3’ splice site. The topological changes induced by PUF60 dimerization upon nucleic acid binding are likely to be important for processing functional complexes at the 3’ splice site, and may be of importance in weak 3’ splice site recognition and splicing.

### Recognition of degenerate PPT in eukaryotes

Recognizing various degenerate PPT sequences in eukaryotic genome is a critical task for splicing factors like U2AF65 and PUF60 that are widely used. U2AF65 and PUF60 need to possess flexibility and cannot be overly stringent with respect to binding specificity. The structure and biophysical studies have suggested that U2AF65 may apply several layers of flexibility to recognize the often degenerate PPTs: (1) flexible side chain conformation on its RNA-binding surface [11,37], (2) coordinating with bound water molecules to form its RNA-binding surface [11], and (3) flexible inter-RRM linker to confer plasticity of the relative arrangement of multiple RRMs [38].

It was found that PUF60 prefers to bind to poly(U) over poly(A), poly(C) or poly(G), based on their respective abilities to compete against pyrimidine tract RNA [12]. Moreover, according to the same study, the affinity for poly(CU) is similar to that of poly(U) and the affinity of poly(GU) appears even greater. The crystal structure of the PUF60 RRMs-dAdML3' complex demonstrates that preference structurally, as UU is observed bound to one side and UG is bound to the other. Therefore, it is the 5'-UG-3' in the poly(GU) that attracts the PUF60 tandem RRM domains. The affinity for poly(CU) is consistent with the observation of cytosine in the first position on one side of the dimer interface in the FIR:FUSE structure. A possibility is that PUF60 may accommodate the needs for both sequence specificity and flexibility by adopting a short, but highly specific binding pocket.

An interesting example about the relevance of sequence specificity in alternative splicing exists between the polypyrimidine tract binding protein (PTB) and U2AF65. PTB and U2AF65 compete for binding on PPT. U2AF65 prefers binding to U-tracts, and can exert its flexibility to adapt to most PPT sequences. On the other hand, PTB prefers CU-rich PPTs. The sequence selectivity may correlate to the fact that several C to U changes in some CU-rich PPTs can remove the repression by PTB and make the alternative exons constitutive [39–42].

### Nucleotide recognition and base specificity

The tandem RRM construct used in the co-crystallization with dAdML3' is the same as was co-crystallized with DNA of the FUSE enhancer element of the *c-myc* gene in a previous study [36]. The fact that this construct recognizes both RNA and DNA reflects flexibility of base recognition. Although the primary interaction of the protein with these single-stranded nucleic acid ligands is with the bases and not the backbone, the sequences recognized in dAdML3' are quite different from those recognized in FUSE. In FUSE, the protein recognizes CG on one side of the dimer, and AT on the other. However, the two Lys-201 residues do not cross over the subunit interface when bound to FUSE as they do when bound to dAdML3'. (Lys-201 corresponds to Lys-184 in the FIR-FUSE structure because 17 residues are missing in full-length FIR relative to PUF60 due to alternative splicing. However, the nucleic acid binding-domain construct used in both studies immediately follows those 17 residues, and therefore the constructs are identical. In this paper, the PUF60 residue numbering is used to be consistent.) Rather, when bound to FUSE, Lys-201 from one subunit, extends outward towards the phosphodiester backbone, presumably due to electrostatic attraction. This causes the other Lys-201 residue to fold into the dimer interface, as it is repelled electrostatically by the other Lys-201 residue. This allows the Lys-201 residue to participate in the formation of the dimeric interface, promoting dimer formation.

In the case of dAdML3', dimer formation is also promoted by the oligonucleotide, however, it is by crossing of the Lys-201 residues. Lys-201 from one subunit hydrogen bonds with the uracil at the first position on one side of the dimer, reaching across the dimeric interface as it participates in this interaction. However, if thymine, which also possesses a carbonyl at the 4-position, were present instead of uracil, the methyl group at position 5 would be within 2.8 Å from the epsilon-amino group of Lys-201. Although this is sterically possible, it is not a favorable interaction. This may be the explanation for the preference of cytosine or adenine in the first position when a DNA sequence is used. However, the presence of uracil in RNA provides a base that is apparently preferred over the others for binding in the first position. Therefore, absence of uracil changes the sequence specificity.

The structure of PUF60 RRMs was determined in the absence of ligand (S6 and S7 Figs, PDB ID: 5KWQ). Although it was determined that this construct is a monomer in solution [36], the crystal structure, presumably due to the very high concentration in the crystal, revealed a dimer with the Lys-201 residues crossing the subunit interface, similar to that in the dAdML3'-bound structure. Therefore, the dAdML3' sequence takes advantage of an inherent tendency of PUF60 RRMs to dimerize with the binding site Lys-201 residues crossing over to mediate protein dimerization. The protein-protein interactions between the subunits do not involve Lys-201 but rather several other residues (S4 Fig). These residues actually form the dimeric interface, and yield this inherent dimerization tendency. However, in the unbound PUF60 RRMs structure, most of the residues in the dimeric interface are not close enough to hydrogen bond (S7 Fig). Although the dimerization is unrealized at normal protein concentration, the interaction of uracil in the oligonucleotide with Lys-201 adds one more hydrogen bond, which would make dimer formation more energetically favorable. Due to the fact that separate portions of the dAdML3' sequence is recognized by the two subunits, it is possible for there to be a 2:1 protein to nucleic acid ratio even in the absence of dimerization (simply having one PUF60 RRMs monomer bound at the end of the polypyrimidine tract and another PUF60 RRMs monomer bound in the middle of the sequence). However, the dimerization forces the nucleic acid to take the looped topology that it adopts.

### PUF60 interacts with SF1 to recognize the 3’ splice site region

The 3’ ss region contains several features including the BPS, the PPT, and the 3’ AG cleavage site. The BPS is a critical determinant for 3’ss selection. Base substitution in the BPS alters 3’ ss selection *in vivo* [43]. SF1 is the major factor to recognize the BPS, using its K-homology (KH) motif to perform sequence specific recognition of the BPS, and its accessory modules to assist the recognition, probably through interaction with the phosphodiester backbone of RNA [44].

SF1 also possesses an N-terminal UHM Ligand Motif (ULM) and can interact with UHM-proteins such as U2AF65 and PUF60 [45]. Through the UHM-ULM interaction, U2AF65 and SF1 cooperatively facilitate recognition of the BPS [8]. The studies reported herein reveal that PUF60 also uses its UHM to interact with SF1, and forms a ternary complex with both SF1 and pre-mRNA (Fig 10). This emphasizes the common feature between U2AF65 and PUF60. However, a major difference between the domain structures of U2AF65 and PUF60 is the extended linker between RRM2 and UHM in PUF60 compared to U2AF65. Considering the looped structure of pre-mRNA that may be induced by PUF60, we hypothesize that the extended linker in PUF60 might confer flexibility for its UHM to search and reach SF1, which may be positioned more distantly due to the looped pre-mRNA structure.

## Conclusion

The current literature suggests that PUF60 and U2AF65 use different strategies to affect pre-mRNA structure to favor splicing, and recognize degenerate PPT sequences. Our study defines the sequence specificity of the alternative splicing factor PUF60 at the 3’ splice site. This information may be important for understanding the molecular mechanism by which alternative and constitutive splicing factors differentiate pre-mRNA 3’ splice sites.

## Supporting information

S1 Fig Electron density of dAdML3’-BrG4 bound to PUF60 RRMs. Brominating Guanine-4 on the C8 atom has no effect on the binding mode of dAdML3’ to PUF60 RRMs. Simulated annealing omit maps, contoured at 3σ, in which all nucleotides (A,B) or the nucleotides bound in the second position of each subunit (C,D) were omitted from the electron density calculations. (A) U-17 bound to subunit A. (B) Uracil from the Poly-U region bound to the first position on subunit B. (C) G-18 bound to subunit A (position 2). (D) the poly-U tract uracil that is bound in the second position on subunit B.
(DOCX)

S2 Fig Electron density of dAdML3’-BrG18 bound to PUF60 RRMs. ssBrominating Guanine-18 on the C8 atom changes the nucleotides bound to PUF60 RRMs. Simulated annealing omit maps were calculated, excluding all nucleotides from the map calculation. The maps are contoured at 3σ. (A) Two uridines bound to subunit A are clearly displayed by the electron density. (B) The same uridines are shown from a different angle to highlight the quality of the electron density even on the second-position uracil, which clearly defines it. (C) Two uridines are also bound to subunit B.
(DOCX)

S3 Fig Two uracil bases are bound on both sides of the dimer when G-18 is brominated. Shown are simulated annealing omit maps, in which only the second position nucleotides were omitted. The electron density is clearer after the first-position nucleotides are added to the model. The map contours are 3σ. (A) Uracil bound in the second position to subunit A (U-12). (B) Uracil from the poly-pyrimidine tract (PPT) bound in the second position to subunit B.
(DOCX)

S4 Fig Dimeric interface interactions in the dAdML3’/ PUF60 RRMs complex. The subunit-subunit interactions are very similar to those observed in the complex of the same dimer with FUSE DNA (23). Hydrogen bonds are represented by dashed lines.
(DOCX)

S5 Fig PUF60 does not dimerize in the absence of dAdML3’. SEC-LS analysis of the PUF60 RRMs+UHM apo-protein. Each data point represents an average molecular weight measured at the apex of an eluent peak of the complex at a certain concentration. Expected MW (47970.7 Da) is indicated by the dashed line, with its expected 5% deviation shown in dotted lines. The dimensions of the X- and Y-axes are intentionally kept identical as in Fig 8A in the main text for comparison.
(DOCX)

S6 Fig Crystal structure of unbound PUF60. (A) The structure of PUF60 RRMs in the absence of oligonucleotide was determined to 2.8 Å. A dimer is observed in the crystal structure, although the protein is monomeric in solution when unbound. (B) The dimer interface observed in the crystal structure of unbound PUF60 RRMs is fairly similar to that seen in the dAdML3’-bound structure, with Lys-201 from each subunit crossing each other to span the dimer interface, suggesting that the dAdML3’ takes advantage of an inherent capability of the protein to dimerize, so that the higher oligomeric state is induced in solution in the presence of the oligonucleotide.
(DOCX)

S7 Fig Dimerization interface found in the crystal structure of PUF60 RRMs in the absence of nucleotide. ssDimerization of unbound PUF60 RRMs is not observed in solution, and therefore must be due to high concentration of protein in the crystal. The similarity of the dimeric interface of the unbound protein to that found when the protein is bound to dAdML3’ indicates that the propensity for dimerization is inherent in the protein before it encounters nucleic acid, and the nucleic acid enhances this propensity to dimerize such that dimerization can occur at lower protein concentration in the presence of nucleic acid. None of the residues in the dimeric interface are close enough for hydrogen bonding, except for the amide nitrogen of each Val-202 donating a hydrogen bond to the carbonyl oxygen of Val-202 of the opposite subunit.
(DOCX)

S1 Table Crystallographic statistics. (DOCX)

S2 Table Hydrogen bond distances between PUF60 RRMs and dAdML3’. (DOCX)

S1 File (PDF)

S2 File (PDF)

S3 File (PDF)

S4 File (PDF)

S5 File (XLS)

S6 File (XLS)

S7 File (XLS)

S8 File (XLS)

## References

[1] Licatalosi DD, Darnell RB. RNA processing and its regulation: global insights into biological networks. Nat Rev Genet. 2010;11(1):75–87. 10.1038/nrg2673 . 20019688 PMC3229837

[2] Smith CW, Valcarcel J. Alternative pre-mRNA splicing: the logic of combinatorial control. Trends Biochem Sci. 2000;25(8):381–8. 10.1016/s0968-0004(00)01604-2 . 10916158

[3] Black DL. Mechanisms of alternative pre-messenger RNA splicing. Annu Rev Biochem. 2003;72:291–336. 10.1146/annurev.biochem.72.121801.161720 12626338

[4] Chen M, Manley JL. Mechanisms of alternative splicing regulation: insights from molecular and genomics approaches. Nat Rev Mol Cell Biol. 2009;10(11):741–54. 10.1038/nrm2777 . 19773805 PMC2958924

[5] Baralle D, Lucassen A, Buratti E. Missed threads. The impact of pre-mRNA splicing defects on clinical practice. EMBO Rep. 2009;10(8):810–6. 10.1038/embor.2009.170 . 19648957 PMC2726684

[6] Khoo B, Krainer AR. Splicing therapeutics in SMN2 and APOB. Curr Opin Mol Ther. 2009;11(2):108–15. . 19330716 PMC3140428

[7] Zamore PD, Patton JG, Green MR. Cloning and domain structure of the mammalian splicing factor U2AF. Nature. 1992;355(6361):609–14. 10.1038/355609a0 . 1538748

[8] Berglund JA, Abovich N, Rosbash M. A cooperative interaction between U2AF65 and mBBP/SF1 facilitates branchpoint region recognition. Genes Dev. 1998;12(6):858–67. . 9512519 10.1101/gad.12.6.858 PMC316625

[9] Kent OA, Reayi A, Foong L, Chilibeck KA, MacMillan AM. Structuring of the 3' splice site by U2AF65. J Biol Chem. 2003;278(50):50572–7. . 14506271 10.1074/jbc.M307976200

[10] Guth S, Tange TO, Kellenberger E, Valcarcel J. Dual function for U2AF(35) in AG-dependent pre-mRNA splicing. Mol Cell Biol. 2001;21(22):7673–81. 10.1128/MCB.21.22.7673-7681.2001 . 11604503 PMC99938

[11] Sickmier EA, Frato KE, Shen H, Paranawithana SR, Green MR, Kielkopf CL. Structural basis for polypyrimidine tract recognition by the essential pre-mRNA splicing factor U2AF65. Mol Cell. 2006;23(1):49–59. 10.1016/j.molcel.2006.05.025 . 16818232 PMC2043114

[12] Page-McCaw PS, Amonlirdviman K, Sharp PA. PUF60: a novel U2AF65-related splicing activity. RNA. 1999;5(12):1548–60. 10.1017/s1355838299991938 . 10606266 PMC1369877

[13] Poleev A, Hartmann A, Stamm S. A trans-acting factor, isolated by the three-hybrid system, that influences alternative splicing of the amyloid precursor protein minigene. Eur J Biochem. 2000;267(13):4002–10. 10.1046/j.1432-1327.2000.01431.x . 10866799

[14] Hastings ML, Allemand E, Duelli DM, Myers MP, Krainer AR. Control of pre-mRNA splicing by the general splicing factors PUF60 and U2AF(65). PLoS One. 2007;2(6):e538 10.1371/journal.pone.0000538 . 17579712 PMC1888729

[15] Van Buskirk C, Schupbach T. Half pint regulates alternative splice site selection in Drosophila. Dev Cell. 2002;2(3):343–53. 10.1016/s1534-5807(02)00128-4 . 11879639

[16] Verheij JB, de Munnik SA, Dijkhuizen T, de Leeuw N, Olde Weghuis D, van den Hoek GJ, et al An 8.35 Mb overlapping interstitial deletion of 8q24 in two patients with coloboma, congenital heart defect, limb abnormalities, psychomotor retardation and convulsions. Eur J Med Genet. 2009;52(5):353–7. 10.1016/j.ejmg.2009.05.006 . 19464398

[17] Dauber A, Golzio C, Guenot C, Jodelka FM, Kibaek M, Kjaergaard S, et al SCRIB and PUF60 are primary drivers of the multisystemic phenotypes of the 8q24.3 copy-number variant. Am J Hum Genet. 2013;93(5):798–811. 10.1016/j.ajhg.2013.09.010 . 24140112 PMC3824129

[18] Wells C, Spaggiari E, Malan V, Stirnemann JJ, Attie-Bitach T, Ville Y, et al First fetal case of the 8q24.3 contiguous genes syndrome. Am J Med Genet A. 2016;170A(1):239–42. 10.1002/ajmg.a.37411 . 26437074

[19] Santos-Simarro F, Vallespin E, Del Pozo A, Ibanez K, Silla JC, Fernandez L, et al Eye coloboma and complex cardiac malformations belong to the clinical spectrum of PUF60 variants. Clin Genet. 2017 10.1111/cge.12965 . 28074499

[20] El Chehadeh S, Kerstjens-Frederikse WS, Thevenon J, Kuentz P, Bruel AL, Thauvin-Robinet C, et al Dominant variants in the splicing factor PUF60 cause a recognizable syndrome with intellectual disability, heart defects and short stature. Eur J Hum Genet. 2017;25:43–51. Epub Nov 02, 2016. 10.1038/ejhg.2016.133 . 27804958 PMC5159768

[21] Low KJ, Ansari M, Abou Jamra R, Clarke A, El Chehadeh S, FitzPatrick DR, et al PUF60 variants cause a syndrome of ID, short stature, microcephaly, coloboma, craniofacial, cardiac, renal and spinal features. Eur J Hum Genet. 2017;25(5):552–9. 10.1038/ejhg.2017.27 . 28327570 PMC5392357

[22] Mishler DM, Christ AB, Steitz JA. Flexibility in the site of exon junction complex deposition revealed by functional group and RNA secondary structure alterations in the splicing substrate. RNA. 2008;14(12):2657–70. 10.1261/rna.1312808 . 18952819 PMC2590960

[23] Folta-Stogniew E, Williams KR. Determination of molecular masses of proteins in solution: Implementation of an HPLC size exclusion chromatography and laser light scattering service in a core laboratory. J Biomol Tech. 1999;10(2):51–63. . 19499008 PMC2291588

[24] Otwinowski Z, Minor W, et al Processing of X-ray diffraction data collected in oscillation mode. Methods Enzymol. 1997;276:307–26. . 27754618 10.1016/S0076-6879(97)76066-X

[25] Kantardjieff KA, Rupp B. Matthews coefficient probabilities: Improved estimates for unit cell contents of proteins, DNA, and protein-nucleic acid complex crystals. Protein Sci. 2003;12(9):1865–71. 10.1110/ps.0350503 . 12930986 PMC2323984

[26] Matthews BW. Solvent content of protein crystals. J Mol Biol. 1968;33(2):491–7. 10.1016/0022-2836(68)90205-2 . 5700707

[27] McCoy AJ, Grosse-Kunstleve RW, Adams PD, Winn MD, Storoni LC, Read RJ. Phaser crystallographic software. J Appl Crystallogr. 2007;40(Pt 4):658–74. 10.1107/S0021889807021206 . 19461840 PMC2483472

[28] Winn MD, Ballard CC, Cowtan KD, Dodson EJ, Emsley P, Evans PR, et al Overview of the CCP4 suite and current developments. Acta Crystallogr D Biol Crystallogr. 2011;67(Pt 4):235–42. 10.1107/S0907444910045749 . 21460441 PMC3069738

[29] Vagin A, Teplyakov A. MOLREP: an automated program for molecular replacement. Journal of Applied Crystallography. 1997;30:1022–5.

[30] Brunger AT, Adams PD, Clore GM, DeLano WL, Gros P, Grosse-Kunstleve RW, et al Crystallography & NMR system: A new software suite for macromolecular structure determination. Acta Crystallogr D Biol Crystallogr. 1998;54(Pt 5):905–21. 10.1107/s0907444998003254 . 9757107

[31] Brunger AT. Version 1.2 of the Crystallography and NMR system. Nat Protoc. 2007;2(11):2728–33. 10.1038/nprot.2007.406 . 18007608

[32] Murshudov GN, Vagin AA, Dodson EJ. Refinement of macromolecular structures by the maximum-likelihood method. Acta Crystallogr D Biol Crystallogr. 1997;53(Pt 3):240–55. 10.1107/S0907444996012255 . 15299926

[33] Jones TA, Zou JY, Cowan SW, Kjeldgaard M. Improved methods for building protein models in electron density maps and the location of errors in these models. Acta Crystallogr A. 1991;47 (Pt 2):110–9. 10.1107/s0108767390010224 . 2025413

[34] Emsley P, Lohkamp B, Scott WG, Cowtan K. Features and development of Coot. Acta Crystallogr D Biol Crystallogr. 2010;66(Pt 4):486–501. 10.1107/S0907444910007493 . 20383002 PMC2852313

[35] Morin A, Eisenbraun B, Key J, Sanschagrin PC, Timony MA, Ottaviano M, et al Collaboration gets the most out of software. Elife. 2013;2:e01456 10.7554/eLife.01456 . 24040512 PMC3771563

[36] Crichlow GV, Zhou H, Hsiao HH, Frederick KB, Debrosse M, Yang Y, et al Dimerization of FIR upon FUSE DNA binding suggests a mechanism of c-myc inhibition. EMBO J. 2008;27(1):277–89. 10.1038/sj.emboj.7601936 . 18059478 PMC2206118

[37] Thickman KR, Sickmier EA, Kielkopf CL. Alternative conformations at the RNA-binding surface of the N-terminal U2AF(65) RNA recognition motif. J Mol Biol. 2007;366(3):703–10. 10.1016/j.jmb.2006.11.077 . 17188295 PMC1828206

[38] Jenkins JL, Shen H, Green MR, Kielkopf CL. Solution conformation and thermodynamic characteristics of RNA binding by the splicing factor U2AF65. J Biol Chem. 2008;283(48):33641–9. 10.1074/jbc.M806297200 . 18842594 PMC2586248

[39] Clery A, Blatter M, Allain FH. RNA recognition motifs: boring? Not quite. Curr Opin Struct Biol. 2008;18(3):290–8. 10.1016/j.sbi.2008.04.002 . 18515081

[40] Singh R, Valcarcel J, Green MR. Distinct binding specificities and functions of higher eukaryotic polypyrimidine tract-binding proteins. Science. 1995;268(5214):1173–6. 10.1126/science.7761834 . 7761834

[41] Chan RC, Black DL. Conserved intron elements repress splicing of a neuron-specific c-src exon in vitro. Mol Cell Biol. 1997;17(5):2970 10.1128/mcb.17.5.2970 . 9111368 PMC232148

[42] Gromak N, Matlin AJ, Cooper TA, Smith CW. Antagonistic regulation of alpha-actinin alternative splicing by CELF proteins and polypyrimidine tract binding protein. RNA. 2003;9(4):443–56. 10.1261/rna.2191903 . 12649496 PMC1370411

[43] Reed R, Maniatis T. The role of the mammalian branchpoint sequence in pre-mRNA splicing. Genes Dev. 1988;2(10):1268–76. 10.1101/gad.2.10.1268 . 3060403

[44] Berglund JA, Fleming ML, Rosbash M. The KH domain of the branchpoint sequence binding protein determines specificity for the pre-mRNA branchpoint sequence. RNA. 1998;4(8):998–1006. 10.1017/s1355838298980499 . 9701290 PMC1369676

[45] Selenko P, Gregorovic G, Sprangers R, Stier G, Rhani Z, Kramer A, et al Structural basis for the molecular recognition between human splicing factors U2AF65 and SF1/mBBP. Mol Cell. 2003;11(4):965–76. 10.1016/s1097-2765(03)00115-1 . 12718882

