# Supplementary figures and images for: Unraveling the mechanism of recognition of the 3’ splice site of the adenovirus major late promoter intron by the alternative splicing factor PUF60
Source: PLoS One. 2020 Nov 30;15(11):e0242725. doi: 10.1371/journal.pone.0242725 (PMC7703929; doi:10.1371/journal.pone.0242725)

**S1 Fig**


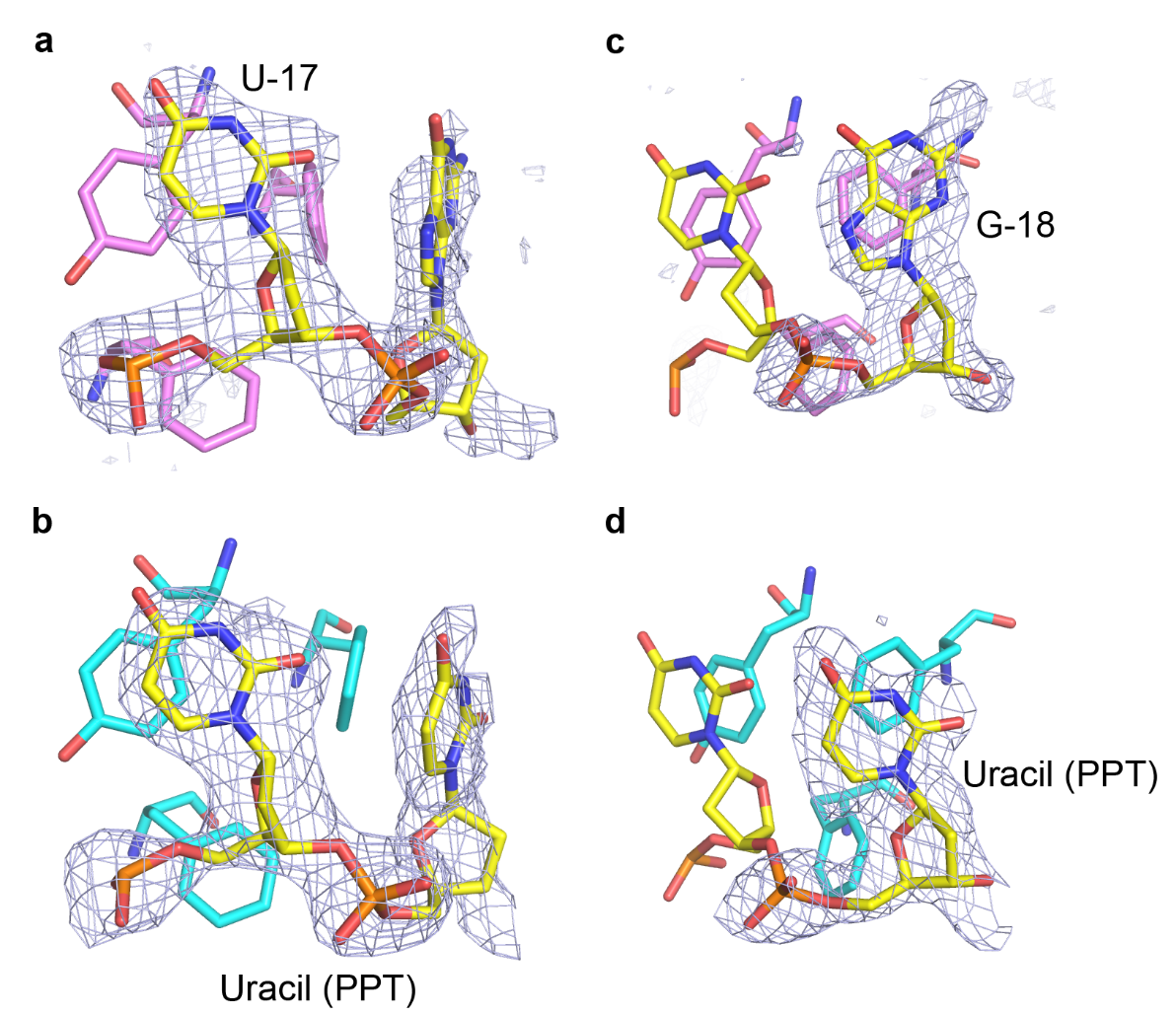

Supplement: S1 Fig — Brominating Guanine-4 on the C8 atom has no effect on the binding mode of dAdML3’ to PUF60 RRMs. Simulated annealing omit maps, contoured at 3σ, in which all nucleotides (A,B) or the nucleotides bound in the second position of each subunit (C,D) were omitted from the electron density calculations. (A) U-17 bound to subunit A. (B) Uracil from the Poly-U region bound to the first position on subunit B. (C) G-18 bound to subunit A (position 2). (D) the poly-U tract uracil that is bound in the second position on subunit B. (DOCX) [file pone.0242725.s001.docx]

**S2 Fig**


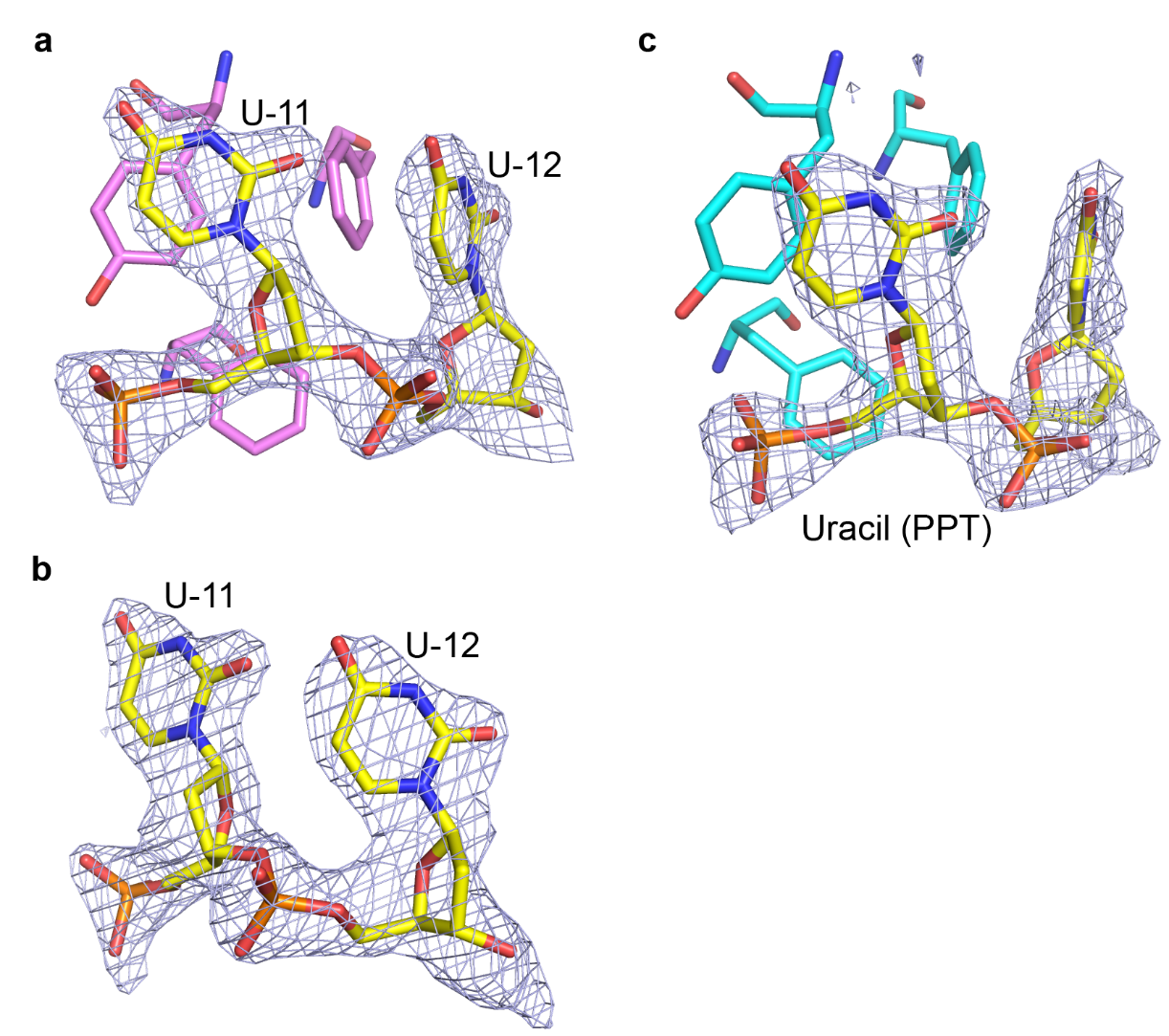

Supplement: S2 Fig — ssBrominating Guanine-18 on the C8 atom changes the nucleotides bound to PUF60 RRMs. Simulated annealing omit maps were calculated, excluding all nucleotides from the map calculation. The maps are contoured at 3σ. (A) Two uridines bound to subunit A are clearly displayed by the electron density. (B) The same uridines are shown from a different angle to highlight the quality of the electron density even on the second-position uracil, which clearly defines it. (C) Two uridines are also bound to subunit B. (DOCX) [file pone.0242725.s002.docx]

**S3 Fig**


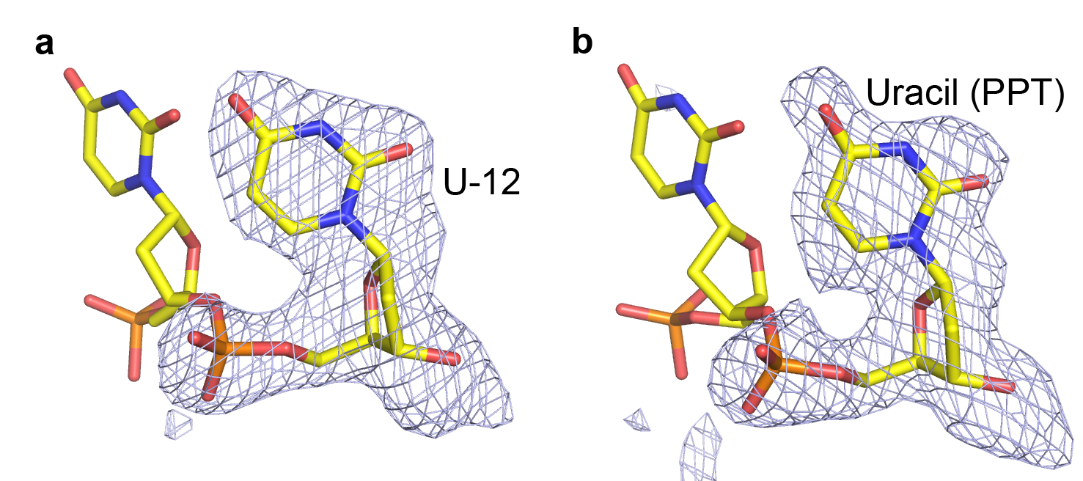

Supplement: S3 Fig — Shown are simulated annealing omit maps, in which only the second position nucleotides were omitted. The electron density is clearer after the first-position nucleotides are added to the model. The map contours are 3σ. (A) Uracil bound in the second position to subunit A (U-12). (B) Uracil from the poly-pyrimidine tract (PPT) bound in the second position to subunit B. (DOCX) [file pone.0242725.s003.docx]

**S4 Fig**

**
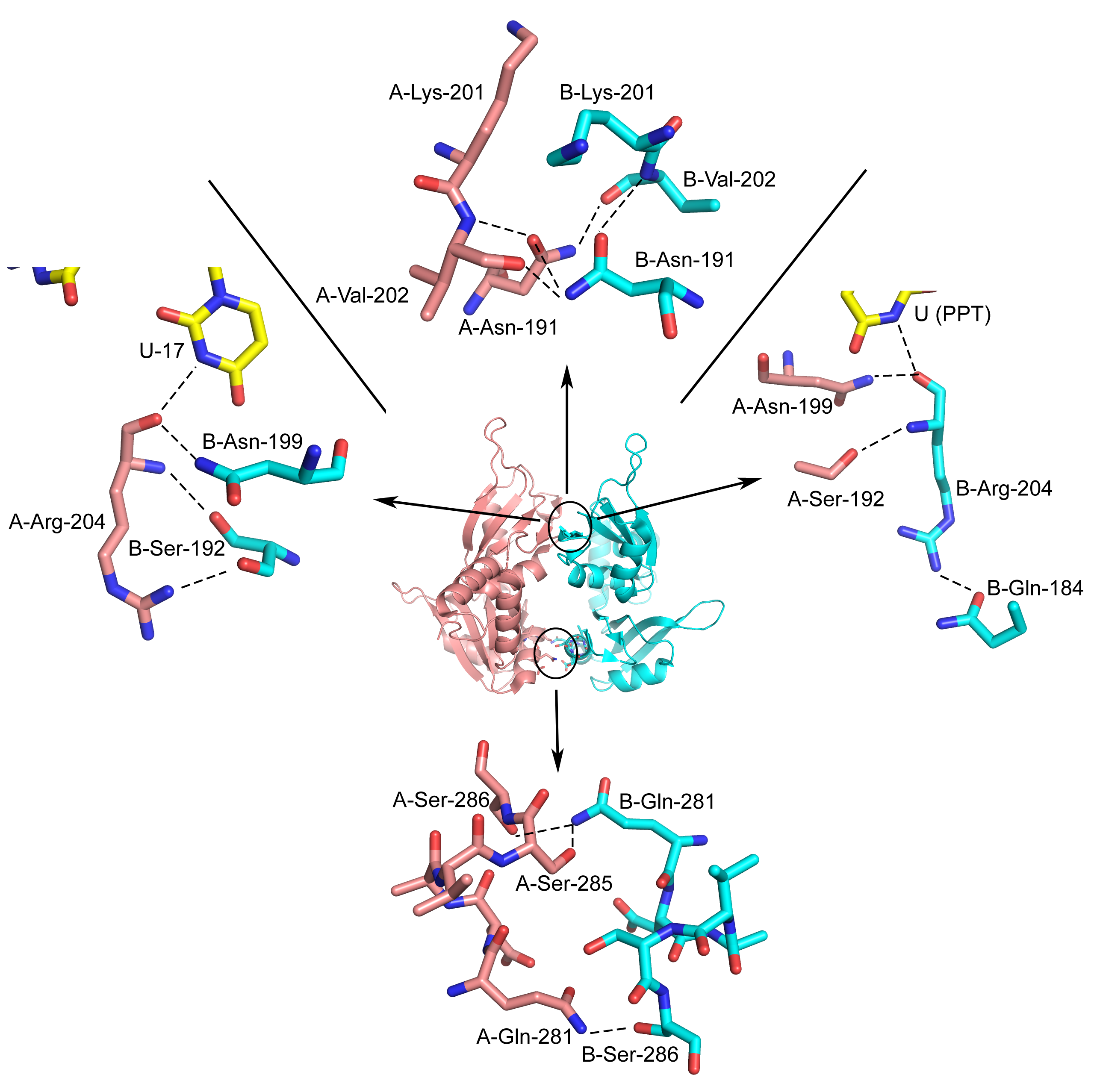
**

Supplement: S4 Fig — The subunit-subunit interactions are very similar to those observed in the complex of the same dimer with FUSE DNA (23). Hydrogen bonds are represented by dashed lines. (DOCX) [file pone.0242725.s004.docx]

**S5 Fig**


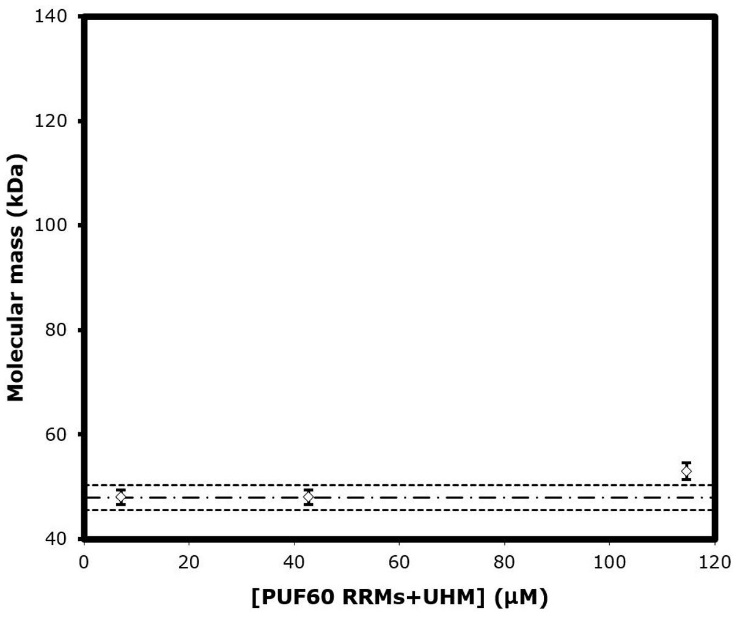

Supplement: S5 Fig — SEC-LS analysis of the PUF60 RRMs+UHM apo-protein. Each data point represents an average molecular weight measured at the apex of an eluent peak of the complex at a certain concentration. Expected MW (47970.7 Da) is indicated by the dashed line, with its expected 5% deviation shown in dotted lines. The dimensions of the X- and Y-axes are intentionally kept identical as in Fig 8A in the main text for comparison. (DOCX) [file pone.0242725.s005.docx]

**S6 Fig**


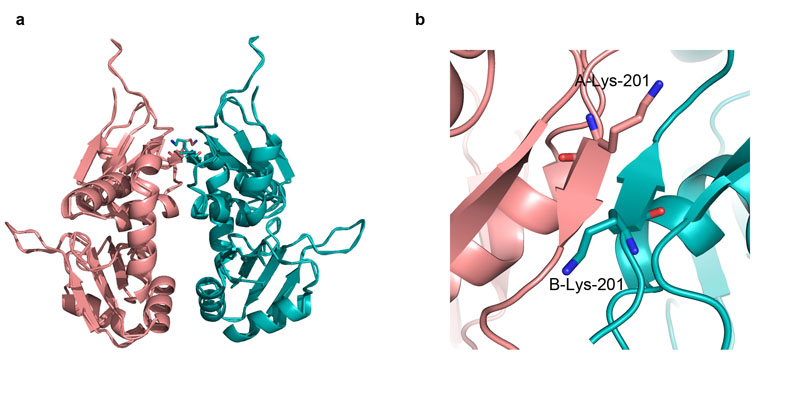

Supplement: S6 Fig — (A) The structure of PUF60 RRMs in the absence of oligonucleotide was determined to 2.8 Å. A dimer is observed in the crystal structure, although the protein is monomeric in solution when unbound. (B) The dimer interface observed in the crystal structure of unbound PUF60 RRMs is fairly similar to that seen in the dAdML3’-bound structure, with Lys-201 from each subunit crossing each other to span the dimer interface, suggesting that the dAdML3’ takes advantage of an inherent capability of the protein to dimerize, so that the higher oligomeric state is induced in solution in the presence of the oligonucleotide. (DOCX) [file pone.0242725.s006.docx]

**S7 Fig**


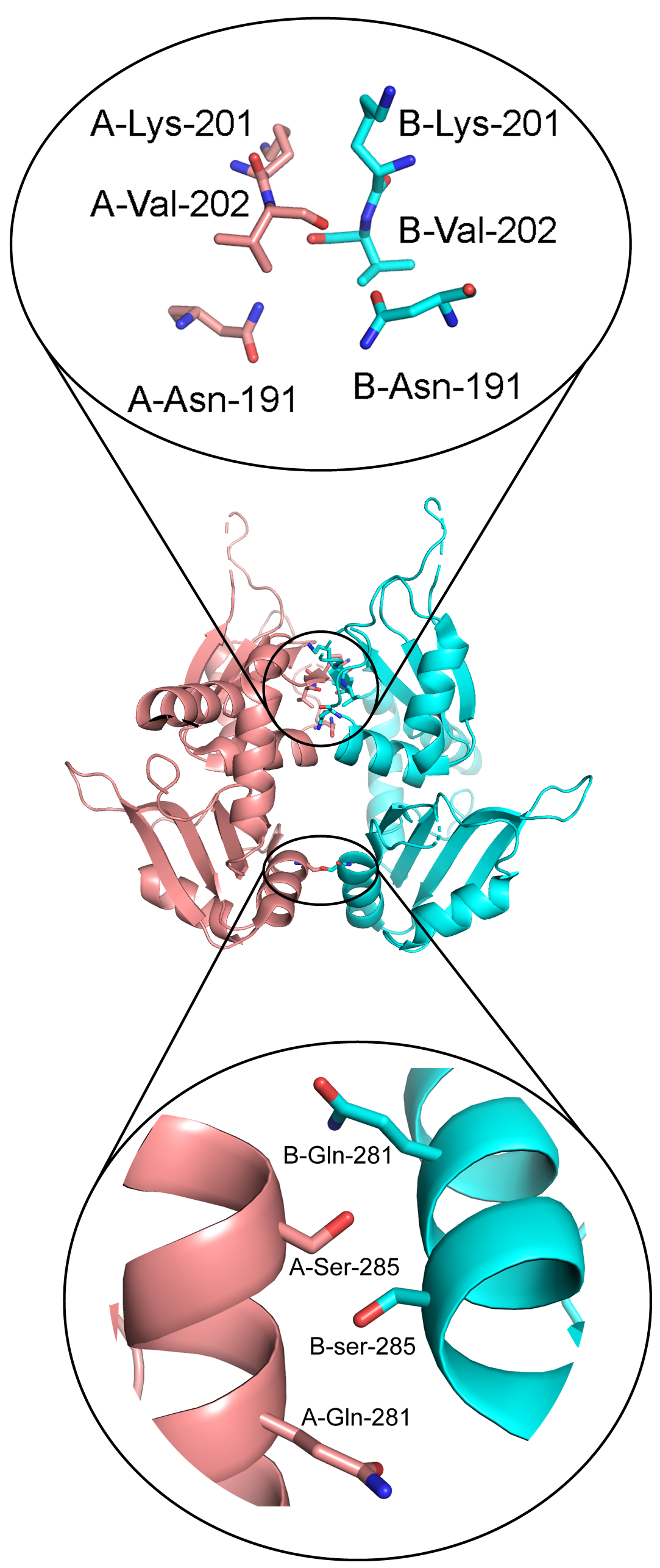

Supplement: S7 Fig — ssDimerization of unbound PUF60 RRMs is not observed in solution, and therefore must be due to high concentration of protein in the crystal. The similarity of the dimeric interface of the unbound protein to that found when the protein is bound to dAdML3’ indicates that the propensity for dimerization is inherent in the protein before it encounters nucleic acid, and the nucleic acid enhances this propensity to dimerize such that dimerization can occur at lower protein concentration in the presence of nucleic acid. None of the residues in the dimeric interface are close enough for hydrogen bonding, except for the amide nitrogen of each Val-202 donating a hydrogen bond to the carbonyl oxygen of Val-202 of the opposite subunit. (DOCX) [file pone.0242725.s007.docx]
