# Supplementary material for: Unraveling the mechanism of recognition of the 3’ splice site of the adenovirus major late promoter intron by the alternative splicing factor PUF60
Source: PLoS One. 2020 Nov 30;15(11):e0242725. doi: 10.1371/journal.pone.0242725 (PMC7703929; doi:10.1371/journal.pone.0242725)
Supplement: S1 Table — (DOCX) [file pone.0242725.s008.docx]

**S1** **Table**. **Crystallographic Statistics**

|  | Wild-type dAdML3’ | G4-brominated | G18-brominated | unbound |
| --- | --- | --- | --- | --- |
| Wavelength (Å) | 0.9795 | 0.9769 | 0.9769 | 1.5418 |
| Unique refl. (total refl.) | 26419 (150999) | 21264 (120900) | 28004 (175272) | 11918 (32561) |
| Resolution (Å) (highest shell) | 30-1.95 (2.00-1.95) | 30-2.10 (2.15-2.10) | 30-1.90 (1.94-1.90) | 50-2.50 (2.59-2.50) |
| R_merge_ (highest shell) | 0.059 (0.452) | 0.099 (0.418) | 0.144 (0.526) | 0.071 (0.745) |
| <I>/<sigma> (highest shell) | 31.6 (3.3) | 14.1 (4.3) | 9.34 (4.1) | 12.7 (3.01)* |
| Completeness, % (highest shell) | 99.7 (100.0) | 99.3 (100.0) | 99.9 (100.0) | 99.1 (92.8) |
| Space group | P3_1_ | P3_1_ | P3_1_ | P3_1_ |
| Unit cell | a=b=62.44 Å, c=83.33 Å;  α=β=90º, γ=120º | a=b=62.43 Å, c=83.31 Å;  α=β=90º, γ=120º | a=b=62.59 Å, c=83.18 Å;  α=β=90º, γ=120º | a=b=61.83 Å, c=80.41 Å; α=β=90º, γ=120º |
|  |  |  |  |  |
|  |  |  |  |  |
| **Refinement** |  |  |  |  |
| Resolution (Å) | 1.95 | 2.10 | 1.90 | 2.80 |
| R_work_/R_free_ | 19.6% / 23.2% | 21.9% / 23.8% | 21.1% / 22.6% | 23.9% / 25.6% |
| r.m.s.d. bond lengths (Å) | 0.006 | 0.009 | 0.007 | 0.014 |
| r.m.s.d. bond angles (deg) | 1.34 | 1.49 | 1.40 | 1.70 |
| Average B-factors (Å^2^) |  |  |  |  |
| Total | 34.49 | 43.68 | 39.41 | 49.43 |
| PUF60 RRMs (chain A) | 32.88 | 42.91 | 38.69 | 49.41 |
| PUF60 RRMs (chain B) | 32.76 | 42.58 | 38.38 | 49.45 |
| dAdML3’ (chain C) | 67.20 | 75.17 | 65.00 |  |
| Water | 44.03 | 46.32 | 44.82 |  |
| Chloride ion |  | 99.16 |  |  |

*value in parentheses represents <I>/<sigma> from 2.96-2.82 Å for the unbound protein crystal, as map calculation and refinement were only performed to 2.8 Å.
