# Supplementary material for: Unraveling the mechanism of recognition of the 3’ splice site of the adenovirus major late promoter intron by the alternative splicing factor PUF60
Source: PLoS One. 2020 Nov 30;15(11):e0242725. doi: 10.1371/journal.pone.0242725 (PMC7703929; doi:10.1371/journal.pone.0242725)
Supplement: S2 Table — (DOCX) [file pone.0242725.s009.docx]

**S2 Table.** **Hydrogen Bond Distances between PUF60 RRMs and dAdML3’**

| protein atom (subunit) | nucleic acid atom | distance (Å) |
| --- | --- | --- |
|  |  |  |
| Tyr-132 OH (A) | U-17 OP2 | 2.29 |
| Lys-201 NZ (B) | U-17 O4 | 2.64 |
| Arg-204 O (A) | U-17 N3 | 3.01 |
| Ser-206 N (A) | U-17 O2 | 2.77 |
| Asn-207 N (A) | G-18 O6 | 2.65 |
| Tyr-132 OH (B) | U-28 OP2 | 2.65 |
| Arg-204 O (B) | U-28 N3 | 3.01 |
| Ser-206 N (B) | U-28 O2 | 2.87 |
| Asn-207 N (B) | U-29 O4 | 2.97 |
|  |  |  |
| Lys-276 NZ (symmetry related copy of subunit A) | U-30 OP1 | 2.43 |
|  |  |  |
| *The following pairs of atoms have somewhat close distances but poor angles for hydrogen-bonding*: | | |
| Ser-206 OG (A) | U-17 N3 | 2.96 |
| Ser-206 OG (B) | U-28 O2 | 3.12 |
| Asn-207 ND2 (B) | U-29 O4 | 3.32 |
