# Supplementary material for: Unraveling the mechanism of recognition of the 3’ splice site of the adenovirus major late promoter intron by the alternative splicing factor PUF60
Source: PLoS One. 2020 Nov 30;15(11):e0242725. doi: 10.1371/journal.pone.0242725 (PMC7703929; doi:10.1371/journal.pone.0242725)
Supplement: S3 File — (PDF) [file pone.0242725.s012.pdf]

Full wwPDB X-ray Structure Validation Report (i)

Mar 9, 2018 – 10:48 am GMT

PDB ID	:	5KW6
Title	:	Two Tandem RRM Domains of PUF60 Bound to an AdML Pre-mRNA 3' Splice Site Analogue with a Modified Binding-Site Nucleic Acid Base
Authors	:	Crichlow, G.V.; Hsiao, H.-H.; Albright, R.; Lolis, E.J.; Braddock, D.T.
Deposited on	:	2016-07-15
Resolution	:	1.91 Å(reported)

This is a Full wwPDB X-ray Structure Validation Report for a publicly released PDB entry.

We welcome your comments at *validation@mail.wwpdb.org*
A user guide is available at
https://www.wwpdb.org/validation/2017/XrayValidationReportHelp
with specific help available everywhere you see the (i) symbol.

The following versions of software and data (see references (i)) were used in the production of this report:

MolProbity	:	4.02b-467
Xtriage (Phenix)	:	1.13
EDS	:	trunk30967
Percentile statistics	:	20171227.v01 (using entries in the PDB archive December 27th 2017)
Refmac	:	5.8.0158
CCP4	:	7.0 (Gargrove)
Ideal geometry (proteins)	:	Engh & Huber (2001)
Ideal geometry (DNA, RNA)	:	Parkinson et al. (1996)
Validation Pipeline (wwPDB-VP)	:	trunk30967

1 Overall quality at a glance (i)

The following experimental techniques were used to determine the structure:
X-RAY DIFFRACTION

The reported resolution of this entry is 1.91 Å.

Percentile scores (ranging between 0-100) for global validation metrics of the entry are shown in the following graphic. The table shows the number of entries on which the scores are based.

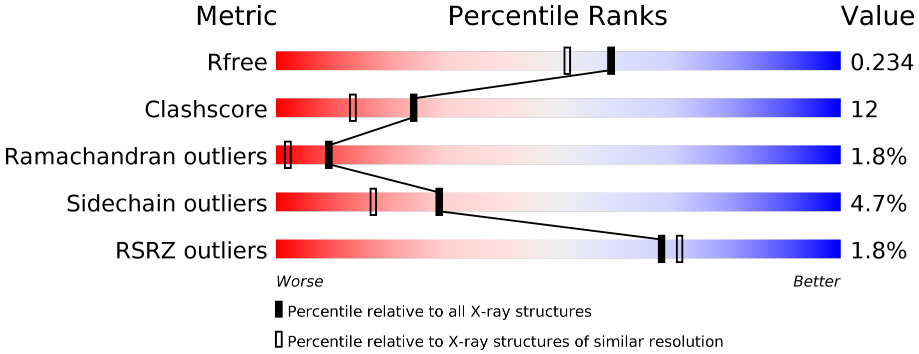

Metric	Whole archive (#Entries)	Similar resolution (#Entries, resolution range(Å))
R_{free}	111664	6904 (1.94-1.90)
Clashscore	122126	7577 (1.94-1.90)
Ramachandran outliers	120053	7491 (1.94-1.90)
Sidechain outliers	120020	7491 (1.94-1.90)
RSRZ outliers	108989	6759 (1.94-1.90)

The table below summarises the geometric issues observed across the polymeric chains and their fit to the electron density. The red, orange, yellow and green segments on the lower bar indicate the fraction of residues that contain outliers for >=3, 2, 1 and 0 types of geometric quality criteria. A grey segment represents the fraction of residues that are not modelled. The numeric value for each fraction is indicated below the corresponding segment, with a dot representing fractions <=5% The upper red bar (where present) indicates the fraction of residues that have poor fit to the electron density. The numeric value is given above the bar.

Mol	Chain	Length	Quality of chain
1	A	216	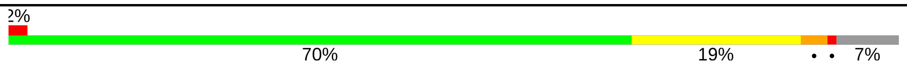
1	B	216	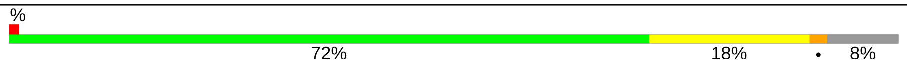
2	C	30	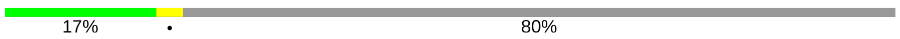

2 Entry composition (i)

There are 3 unique types of molecules in this entry. The entry contains 3263 atoms, of which 0 are hydrogens and 0 are deuteriums.

In the tables below, the ZeroOcc column contains the number of atoms modelled with zero occupancy, the AltConf column contains the number of residues with at least one atom in alternate conformation and the Trace column contains the number of residues modelled with at most 2 atoms.

- Molecule 1 is a protein called Poly(U)-binding-splicing factor PUF60.

Mol	Chain	Residues	Atoms					ZeroOcc	AltConf	Trace
1	A	201	Total 1534	C 971	N 264	O 293	S 6	0	0	0
1	B	198	Total 1524	C 964	N 263	O 289	S 8	0	0	0

There are 40 discrepancies between the modelled and reference sequences:

Chain	Residue	Modelled	Actual	Comment	Reference
A	101	GLY	-	expression tag	UNP Q9UHX1
A	102	SER	-	expression tag	UNP Q9UHX1
A	103	HIS	-	expression tag	UNP Q9UHX1
A	104	MET	-	expression tag	UNP Q9UHX1
A	105	ALA	-	expression tag	UNP Q9UHX1
A	106	SER	-	expression tag	UNP Q9UHX1
A	107	MET	-	expression tag	UNP Q9UHX1
A	108	THR	-	expression tag	UNP Q9UHX1
A	109	GLY	-	expression tag	UNP Q9UHX1
A	110	GLY	-	expression tag	UNP Q9UHX1
A	111	GLN	-	expression tag	UNP Q9UHX1
A	112	GLN	-	expression tag	UNP Q9UHX1
A	113	MET	-	expression tag	UNP Q9UHX1
A	114	GLY	-	expression tag	UNP Q9UHX1
A	115	ARG	-	expression tag	UNP Q9UHX1
A	116	GLY	-	expression tag	UNP Q9UHX1
A	117	SER	-	expression tag	UNP Q9UHX1
A	123	GLY	ARG	engineered mutation	UNP Q9UHX1
A	129	SER	CYS	engineered mutation	UNP Q9UHX1
A	255	ALA	CYS	engineered mutation	UNP Q9UHX1
B	101	GLY	-	expression tag	UNP Q9UHX1
B	102	SER	-	expression tag	UNP Q9UHX1
B	103	HIS	-	expression tag	UNP Q9UHX1
B	104	MET	-	expression tag	UNP Q9UHX1
B	105	ALA	-	expression tag	UNP Q9UHX1

Continued on next page...

Continued from previous page...

Chain	Residue	Modelled	Actual	Comment	Reference
B	106	SER	-	expression tag	UNP Q9UHX1
B	107	MET	-	expression tag	UNP Q9UHX1
B	108	THR	-	expression tag	UNP Q9UHX1
B	109	GLY	-	expression tag	UNP Q9UHX1
B	110	GLY	-	expression tag	UNP Q9UHX1
B	111	GLN	-	expression tag	UNP Q9UHX1
B	112	GLN	-	expression tag	UNP Q9UHX1
B	113	MET	-	expression tag	UNP Q9UHX1
B	114	GLY	-	expression tag	UNP Q9UHX1
B	115	ARG	-	expression tag	UNP Q9UHX1
B	116	GLY	-	expression tag	UNP Q9UHX1
B	117	SER	-	expression tag	UNP Q9UHX1
B	123	GLY	ARG	engineered mutation	UNP Q9UHX1
B	129	SER	CYS	engineered mutation	UNP Q9UHX1
B	255	ALA	CYS	engineered mutation	UNP Q9UHX1

- Molecule 2 is a DNA chain called DNA (30-MER).

Mol	Chain	Residues	Atoms					ZeroOcc	AltConf	Trace
2	C	6	Total	C	N	O	P	0	0	2
			78	36	8	30	4			

- Molecule 3 is water.

Mol	Chain	Residues	Atoms		ZeroOcc	AltConf
3	A	68	Total	O	0	0
			68	68		
3	B	57	Total	O	0	0
			57	57		
3	C	2	Total	O	0	0
			2	2		

3 Residue-property plots (i)

These plots are drawn for all protein, RNA and DNA chains in the entry. The first graphic for a chain summarises the proportions of the various outlier classes displayed in the second graphic. The second graphic shows the sequence view annotated by issues in geometryand electron density. Residues are color-coded according to the number of geometric quality criteria for which they contain at least one outlier: green = 0, yellow = 1, orange = 2 and red = 3 or more. A red dot above a residue indicates a poor fit to the electron density (RSRZ > 2). Stretches of 2 or more consecutive residues without any outlier are shown as a green connector. Residues present in the sample, but not in the model, are shown in grey.

- Molecule 1: Poly(U)-binding-splicing factor PUF60

Chain A:

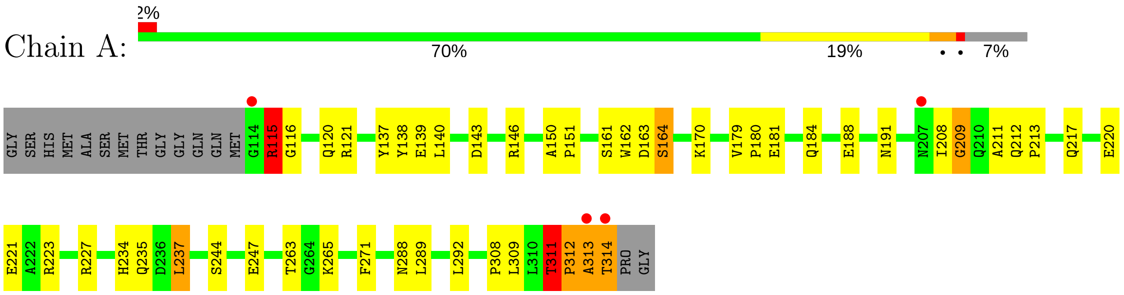

- Molecule 1: Poly(U)-binding-splicing factor PUF60

Chain B:

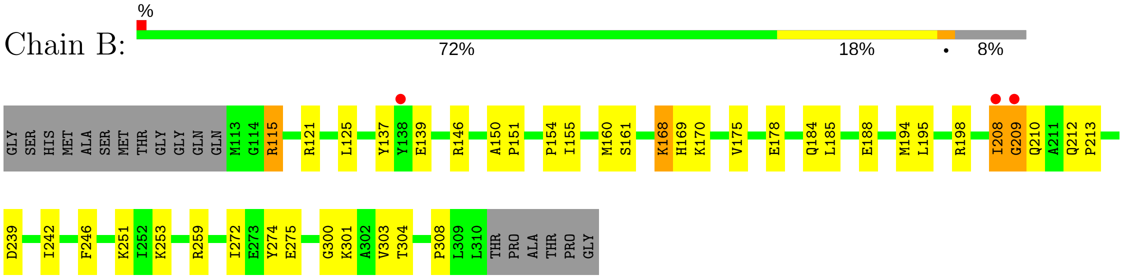

- Molecule 2: DNA (30-MER)

Chain C:

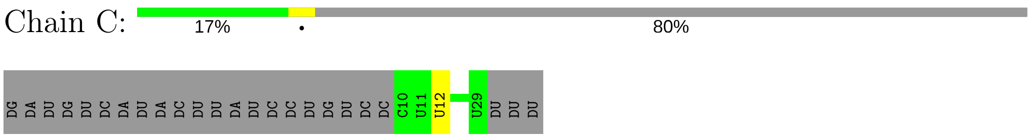

4 Data and refinement statistics

Property	Value	Source
Space group	P 31	Depositor
Cell constants<br>a, b, c, α, β, γ	62.59Å 62.59Å 83.18Å<br>90.00° 90.00° 120.00°	Depositor
Resolution (Å)	29.29 – 1.91<br>29.29 – 1.91	Depositor<br>EDS
% Data completeness<br>(in resolution range)	93.5 (29.29-1.91)<br>93.8 (29.29-1.91)	Depositor<br>EDS
R_{merge}	0.14	Depositor
R_{sym}	(Not available)	Depositor
$< I/\sigma(I) >$ [1]	2.85 (at 1.91Å)	Xtriage
Refinement program	CNS 1.3	Depositor
R, R_{free}	0.211 , 0.226<br>0.220 , 0.234	Depositor<br>DCC
R_{free} test set	1247 reflections (4.70%)	wwPDB-VP
Wilson B-factor (Å^2)	33.0	Xtriage
Anisotropy	0.159	Xtriage
Bulk solvent k_{sol}(e/Å^3), B_{sol}(Å^2)	0.32 , 30.4	EDS
L-test for twinning[2]	$< \|L\| >= 0.51$, $< L^2 >= 0.35$	Xtriage
Estimated twinning fraction	0.024 for -h,-k,l<br>0.348 for h,-h-k,-l<br>0.027 for -k,-h,-l	Xtriage
F_o,F_c correlation	0.96	EDS
Total number of atoms	3263	wwPDB-VP
Average B, all atoms (Å^2)	39.0	wwPDB-VP

Xtriage's analysis on translational NCS is as follows: *The largest off-origin peak in the Patterson function is 4.95% of the height of the origin peak. No significant pseudotranslation is detected.*

[1]Intensities estimated from amplitudes.

[2]Theoretical values of $< |L| >$, $< L^2 >$ for acentric reflections are 0.5, 0.333 respectively for untwinned datasets, and 0.375, 0.2 for perfectly twinned datasets.

5 Model quality (i)

5.1 Standard geometry (i)

The Z score for a bond length (or angle) is the number of standard deviations the observed value is removed from the expected value. A bond length (or angle) with $|Z| > 5$ is considered an outlier worth inspection. RMSZ is the root-mean-square of all Z scores of the bond lengths (or angles).

Mol	Chain	Bond lengths		Bond angles	
		RMSZ	#$\|Z\|$ >5	RMSZ	#$\|Z\|$ >5
1	A	0.39	0/1565	0.64	0/2115
1	B	0.38	0/1554	0.61	0/2095
2	C	0.75	0/84	0.68	0/126
All	All	0.40	0/3203	0.63	0/4336

There are no bond length outliers.

There are no bond angle outliers.

There are no chirality outliers.

There are no planarity outliers.

5.2 Too-close contacts (i)

In the following table, the Non-H and H(model) columns list the number of non-hydrogen atoms and hydrogen atoms in the chain respectively. The H(added) column lists the number of hydrogen atoms added and optimized by MolProbity. The Clashes column lists the number of clashes within the asymmetric unit, whereas Symm-Clashes lists symmetry related clashes.

Mol	Chain	Non-H	H(model)	H(added)	Clashes	Symm-Clashes
1	A	1534	0	1510	41	0
1	B	1524	0	1511	31	0
2	C	78	0	42	1	0
3	A	68	0	0	1	0
3	B	57	0	0	2	0
3	C	2	0	0	0	0
All	All	3263	0	3063	73	0

The all-atom clashscore is defined as the number of clashes found per 1000 atoms (including hydrogen atoms). The all-atom clashscore for this structure is 12.

All (73) close contacts within the same asymmetric unit are listed below, sorted by their clash magnitude.

Atom-1	Atom-2	Interatomic distance (Å)	Clash overlap (Å)
1:B:168:LYS:HE3	1:B:169:HIS:H	1.04	1.13
1:A:311:THR:HB	1:A:312:PRO:HA	1.29	1.09
1:B:168:LYS:HE3	1:B:169:HIS:N	1.81	0.95
1:B:210:GLN:HG2	1:B:210:GLN:O	1.67	0.94
1:B:168:LYS:HE2	1:B:169:HIS:CD2	2.08	0.88
1:B:251:LYS:HE3	1:B:275:GLU:OE2	1.76	0.84
1:A:311:THR:HB	1:A:312:PRO:CA	2.07	0.83
1:A:234:HIS:HB3	1:A:237:LEU:CD2	2.19	0.72
1:A:162:TRP:NE1	1:A:164:SER:HB2	2.05	0.71
1:A:234:HIS:HB3	1:A:237:LEU:HD22	1.70	0.71
1:A:163:ASP:O	1:A:164:SER:HB3	1.94	0.66
1:A:244:SER:O	1:A:247:GLU:HG2	1.96	0.65
1:A:237:LEU:HD21	1:A:292:LEU:CD2	2.26	0.65
1:B:209:GLY:O	1:B:210:GLN:HB3	1.98	0.64
1:A:311:THR:CB	1:A:312:PRO:HA	2.10	0.64
1:B:137:TYR:HA	1:B:198:ARG:CZ	2.28	0.63
1:A:184:GLN:O	1:A:188:GLU:HG3	2.03	0.58
1:B:210:GLN:CG	1:B:210:GLN:O	2.49	0.57
1:B:154:PRO:HG2	1:B:178:GLU:OE2	2.05	0.56
1:A:161:SER:HB2	1:A:170:LYS:HB2	1.88	0.56
1:A:162:TRP:HE1	1:A:164:SER:HB2	1.70	0.56
1:A:292:LEU:HD23	1:A:292:LEU:C	2.26	0.56
1:A:208:ILE:HG23	1:A:211:ALA:HB3	1.86	0.55
1:A:143:ASP:N	1:A:143:ASP:OD1	2.39	0.55
1:A:138:TYR:CE1	1:A:139:GLU:HG2	2.42	0.55
1:A:220:GLU:HG2	1:A:223:ARG:HH21	1.71	0.54
1:A:227:ARG:HD3	3:A:410:HOH:O	2.08	0.54
1:A:312:PRO:O	1:A:313:ALA:HB2	2.06	0.54
1:B:239:ASP:OD1	1:B:259:ARG:NH2	2.34	0.54
1:A:115:ARG:HB3	1:A:217:GLN:HG2	1.88	0.54
1:B:168:LYS:CE	1:B:169:HIS:CD2	2.86	0.53
1:A:179:VAL:HG13	1:A:180:PRO:HD2	1.92	0.52
1:B:121:ARG:O	1:B:125:LEU:HG	2.11	0.51
1:A:237:LEU:HD21	1:A:292:LEU:HD22	1.91	0.51
1:B:146:ARG:HH11	1:B:146:ARG:HB3	1.76	0.51
1:B:184:GLN:O	1:B:188:GLU:HG3	2.12	0.50
1:A:311:THR:CB	1:A:312:PRO:CA	2.83	0.50
1:A:179:VAL:HG12	1:A:181:GLU:H	1.76	0.49
1:A:212:GLN:HB3	1:A:213:PRO:HD3	1.94	0.49
1:B:155:ILE:HD12	1:B:175:VAL:CG1	2.42	0.48
1:A:220:GLU:HG2	1:A:223:ARG:NH2	2.29	0.48
1:B:208:ILE:HG12	1:B:209:GLY:N	2.29	0.48

Continued on next page...

Continued from previous page...

Atom-1	Atom-2	Interatomic distance (Å)	Clash overlap (Å)
1:B:115:ARG:HD3	1:B:115:ARG:HA	1.50	0.47
1:B:242:ILE:HG22	1:B:272:ILE:HD11	1.96	0.47
2:C:12:DU:H6	2:C:12:DU:O5'	2.14	0.47
1:A:217:GLN:HG3	1:A:221:GLU:OE2	2.14	0.47
1:A:179:VAL:CG1	1:A:181:GLU:OE1	2.63	0.47
1:A:163:ASP:O	1:A:164:SER:CB	2.63	0.46
1:A:311:THR:HB	1:A:312:PRO:O	2.16	0.46
1:A:217:GLN:HE21	1:A:221:GLU:HG3	1.80	0.46
1:B:185:LEU:HD21	1:B:300:GLY:HA3	1.97	0.46
1:A:179:VAL:HG11	1:A:271:PHE:HZ	1.82	0.45
1:A:150:ALA:N	1:A:151:PRO:CD	2.80	0.44
1:B:137:TYR:HA	1:B:198:ARG:NE	2.32	0.44
1:A:191:ASN:HB3	3:B:440:HOH:O	2.17	0.44
1:B:121:ARG:NE	1:B:308:PRO:O	2.47	0.44
1:B:212:GLN:N	1:B:213:PRO:HD2	2.34	0.43
1:B:146:ARG:HB3	1:B:146:ARG:NH1	2.33	0.43
1:B:150:ALA:N	1:B:151:PRO:CD	2.82	0.43
1:A:137:TYR:HB3	1:A:140:LEU:HG	2.01	0.42
1:B:210:GLN:N	3:B:401:HOH:O	2.46	0.42
1:A:288:ASN:O	1:A:289:LEU:HB2	2.20	0.42
1:B:246:PHE:HB3	1:B:274:TYR:OH	2.19	0.42
1:B:253:LYS:HE3	1:B:275:GLU:HA	2.01	0.42
1:B:303:VAL:HG23	1:B:304:THR:HG23	2.02	0.42
1:B:161:SER:HB2	1:B:170:LYS:HB2	2.01	0.41
1:A:208:ILE:O	1:A:209:GLY:C	2.58	0.41
1:A:313:ALA:O	1:A:314:THR:HB	2.21	0.41
1:B:251:LYS:HE3	1:B:275:GLU:CD	2.41	0.41
1:A:263:THR:HB	1:A:265:LYS:HG3	2.01	0.41
1:A:116:GLY:HA2	1:A:120:GLN:OE1	2.21	0.40
1:B:208:ILE:O	1:B:209:GLY:C	2.59	0.40
1:A:121:ARG:NE	1:A:308:PRO:O	2.51	0.40

There are no symmetry-related clashes.

5.3 Torsion angles (i)

5.3.1 Protein backbone (i)

In the following table, the Percentiles column shows the percent Ramachandran outliers of the chain as a percentile score with respect to all X-ray entries followed by that with respect to entries of similar resolution.

The Analysed column shows the number of residues for which the backbone conformation was analysed, and the total number of residues.

Mol	Chain	Analysed	Favoured	Allowed	Outliers	Percentiles
1	A	199/216 (92%)	188 (94%)	5 (2%)	6 (3%)	5 0
1	B	196/216 (91%)	191 (97%)	4 (2%)	1 (0%)	31 19
All	All	395/432 (91%)	379 (96%)	9 (2%)	7 (2%)	9 2

All (7) Ramachandran outliers are listed below:

Mol	Chain	Res	Type
1	A	311	THR
1	A	312	PRO
1	A	313	ALA
1	A	115	ARG
1	A	209	GLY
1	B	209	GLY
1	A	164	SER

5.3.2 Protein sidechains (i)

In the following table, the Percentiles column shows the percent sidechain outliers of the chain as a percentile score with respect to all X-ray entries followed by that with respect to entries of similar resolution.

The Analysed column shows the number of residues for which the sidechain conformation was analysed, and the total number of residues.

Mol	Chain	Analysed	Rotameric	Outliers	Percentiles
1	A	158/171 (92%)	151 (96%)	7 (4%)	31 19
1	B	159/171 (93%)	151 (95%)	8 (5%)	27 15
All	All	317/342 (93%)	302 (95%)	15 (5%)	29 17

All (15) residues with a non-rotameric sidechain are listed below:

Mol	Chain	Res	Type
1	A	115	ARG
1	A	146	ARG
1	A	235	GLN
1	A	237	LEU
1	A	309	LEU
1	A	311	THR

Continued on next page...

Continued from previous page...

Mol	Chain	Res	Type
1	A	314	THR
1	B	115	ARG
1	B	139	GLU
1	B	160	MET
1	B	168	LYS
1	B	194	MET
1	B	195	LEU
1	B	208	ILE
1	B	301	LYS

Some sidechains can be flipped to improve hydrogen bonding and reduce clashes. All (3) such sidechains are listed below:

Mol	Chain	Res	Type
1	A	147	GLN
1	A	235	GLN
1	B	295	GLN

5.3.3 RNA ⓘ

There are no RNA molecules in this entry.

5.4 Non-standard residues in protein, DNA, RNA chains ⓘ

There are no non-standard protein/DNA/RNA residues in this entry.

5.5 Carbohydrates ⓘ

There are no carbohydrates in this entry.

5.6 Ligand geometry ⓘ

There are no ligands in this entry.

5.7 Other polymers ⓘ

There are no such residues in this entry.

5.8 Polymer linkage issues (i)

There are no chain breaks in this entry.

6 Fit of model and data (i)

6.1 Protein, DNA and RNA chains (i)

In the following table, the column labelled '#RSRZ> 2' contains the number (and percentage) of RSRZ outliers, followed by percent RSRZ outliers for the chain as percentile scores relative to all X-ray entries and entries of similar resolution. The OWAB column contains the minimum, median, 95th percentile and maximum values of the occupancy-weighted average B-factor per residue. The column labelled 'Q< 0.9' lists the number of (and percentage) of residues with an average occupancy less than 0.9.

Mol	Chain	Analysed	<RSRZ>	#RSRZ>2			OWAB(Å^2)	Q<0.9
1	A	201/216 (93%)	-0.24	4 (1%)	65	68	25, 37, 53, 78	0
1	B	198/216 (91%)	-0.32	3 (1%)	73	76	26, 37, 52, 74	0
2	C	1/30 (3%)	-0.35	0	100	100	71, 71, 71, 71	0
All	All	400/462 (86%)	-0.28	7 (1%)	68	71	25, 37, 53, 78	0

All (7) RSRZ outliers are listed below:

Mol	Chain	Res	Type	RSRZ
1	A	114	GLY	3.7
1	A	314	THR	3.3
1	B	209	GLY	3.3
1	A	313	ALA	2.5
1	B	208	ILE	2.4
1	A	207	ASN	2.2
1	B	138	TYR	2.1

6.2 Non-standard residues in protein, DNA, RNA chains (i)

There are no non-standard protein/DNA/RNA residues in this entry.

6.3 Carbohydrates (i)

There are no carbohydrates in this entry.

6.4 Ligands (i)

There are no ligands in this entry.

6.5 Other polymers (i)

There are no such residues in this entry.
